# Optogenetic Stimulation of the Cardiac Vagus Nerve to Promote Heart Regenerative Repair after Myocardial Infarction

**DOI:** 10.7150/ijbs.89883

**Published:** 2024-03-17

**Authors:** Yuan Han, Xiaomin Wei, Guojun Chen, Enge Shao, Yilin Zhou, Yuqing Li, Zhiwen Xiao, Xiaoran Shi, Hao Zheng, Senlin Huang, Yanmei Chen, Yanbing Wang, Yeshen Zhang, Yulin Liao, Wangjun Liao, Jianping Bin, Yuegang Wang, Xinzhong Li

**Affiliations:** 1 Department of Cardiology, State Key Laboratory of Organ Failure Research, Nanfang Hospital, Southern Medical University, Guangzhou, China.; 2 The Sixth Affiliated Hospital, School of Medicine, South China University of Technology, Foshan, China.; 3 Guangdong Provincial Key Laboratory of Cardiac Function and Microcirculation.; 4 Department of Nosocomial Infection Administration, Zhujiang Hospital, Southern Medical University, Guangzhou, China.; 5 Department of Cardiology, Guangdong Provincial People's Hospital (Guangdong Academy of Medical Sciences), Southern Medical University, Guangzhou, China.; 6 Department of Oncology, Nanfang Hospital, Southern Medical University, Guangzhou, China.

**Keywords:** Optogenetic stimulation, cardiac vagal, heart regeneration, myocardial infarction

## Abstract

**Background:** It had been shown that selective cardiac vagal activation holds great potential for heart regeneration. Optogenetics has clinical translation potential as a novel means of modulating targeted neurons. This study aimed to investigate whether cardiac vagal activation via optogenetics could improve heart regenerative repair after myocardial infarction (MI) and to identify the underlying mechanism.

**Methods:** We used an adeno-associated virus (AAV) as the vector to deliver ChR2, a light-sensitive protein, to the left nodose ganglion (LNG). To assess the effects of the cardiac vagus nerve on cardiomyocyte (CM) proliferation and myocardial regeneration *in vivo,* the light-emitting diode illumination (470 nm) was applied for optogenetic stimulation to perform the gain-of-function experiment and the vagotomy was used as a loss-of-function assay. Finally, sequencing data and molecular biology experiments were analyzed to determine the possible mechanisms by which the cardiac vagus nerve affects myocardial regenerative repair after MI.

**Results:** Absence of cardiac surface vagus nerve after MI was more common in adult hearts with low proliferative capacity, causing a poor prognosis. Gain- and loss-of-function experiments further demonstrated that optogenetic stimulation of the cardiac vagus nerve positively regulated cardiomyocyte (CM) proliferation and myocardial regeneration *in vivo*. More importantly, optogenetic stimulation attenuated ventricular remodeling and improved cardiac function after MI. Further analysis of sequencing results and flow cytometry revealed that cardiac vagal stimulation activated the IL-10/STAT3 pathway and promoted the polarization of cardiac macrophages to the M2 type, resulting in beneficial cardiac regenerative repair after MI.

**Conclusions:** Targeting the cardiac vagus nerve by optogenetic stimulation induced macrophage M2 polarization by activating the IL-10/STAT3 signaling pathway, which obviously optimized the regenerative microenvironment and then improved cardiac function after MI.

## Introduction

Various types of myocardial injuries, including myocardial infarction (MI), myocarditis, cardiomyopathy and heart failure, could result in the loss of cardiomyocytes (CMs), which led to poor myocardial remodeling and deterioration of cardiac function 1. Efforts were being made to explore effective ways of promoting myocardial regeneration to replenish lost CMs 2. According to recent findings, the vagus nerve and its guided reparative inflammation were considered to be important links to nerve-driven regenerative programs, in which the activation of cholinergic neurons releases acetylcholine to act on nicotinic acetylcholine receptors (nAChRs) on macrophages of the immune system, balancing an excessive inflammatory response after MI, which is critical for myocardial repair 3, 4. It had been reported that inhibition of the left vagus nerve through the application of drugs or mechanical means can significantly reduce CM proliferation and impede cardiac regenerative repair 5. These lines of evidence indicated that the vagus nerve was essential for myocardial regeneration, suggesting that cardiac vagal activation may confer a regenerative benefit that would be useful for improving the outcomes of myocardial damage. However, it was still unknown whether cardiac vagal stimulation could induce myocardial regeneration and therefore improve cardiac function after MI, which had important clinical implications because it suggested that peripheral stimulation of the cardiac vagus nerve could achieve cardiac regenerative repair. Indeed, due to the important potential of cardiac vagal activation, targeted stimulation of the cardiac vagus nerve was highly valued as a potential way to achieve regenerative functional repair of the heart.

Optogenetics, a novel technology with the unique combination of high spatial and temporal resolution and cell type specificity, was used to silence or enhance the activity of genetically targeted neurons, for example, selectively activating a desired set of neurons 6. In brief, a synergistic light-sensitive opsin was genetically expressed in targeted cells and activated via illumination with the appropriate wavelength and then induced hyperpolarizing currents, thus activating the targeted cells. Compared with the application of molecular targets such as neuregulin 1 (NRG1) and nerve growth factor (NGF) recombinant proteins 5, optogenetics had better discriminability and could improve the application efficiency. A growing number of studies had shown that optogenetic stimulation (OGS) could exert an important influence on immune inflammation and repair of many organs, including the heart, by modulating autonomic nerve activity. OGS was helpful for addressing the “inflammatory dialog” between psychiatric disorders and cardiovascular diseases in the context of stress 7. A recent study reported that OGS was used to selectively stimulate vagal efferent or afferent fibers to confer kidney protection from ischemia‒reperfusion injury by modulating neuroimmune interactions 8. It had been clarified that M2-type macrophage mediated inflammatory response was required for neonatal heart regeneration, and interleukin-10 (IL-10) could be used to promote the polarization of macrophages towards M2 9. Based on these findings, we hypothesized that specific stimulation of the cardiac vagus nerve by OGS could induce a reparative inflammatory response to promote cardiac regenerative repair, therefore improving the prognosis of MI.

The present study aimed to certify the effect of selective cardiac vagal activation by OGS on cardiac regenerative repair. We showed that optogenetics, a method to modulate targeted neurons, could enhance cardiac vagal activity, therefore promote CM proliferation and angiogenesis, thus effectively decreasing the remodeling area and inproving functional recovery after MI. Furthermore, by analyzing sequencing data and performing molecular biology experiments, we found that vagal stimulation activated the IL-10/STAT3 signaling pathway and improved the regenerative microenvironment involved in M2 macrophages, which was critical for cardiac regenerative repair after MI.

## Materials and Methods

### Ethics statement

The Animal Research Committee of Southern Medical University authorized the animal studies in this investigation, and all methods followed the Institutional Guidelines for Animal Research and the Guide for the Care and Use of Laboratory Animals issued by the US National Institutes of Health (2011) 10. Mice were euthanized by cervical dislocation under deep anesthesia with 5% isoflurane and oxygen inhaled through a nose cone. Euthanasia was confirmed by verifying that the animals did not respond to a firm toe pinch.

### CM isolation and culture

The neonatal CMs used for the *in vitro* experiments were extracted from P1 and P7 C57BL/6 newborn mice purchased from the Southern Medical University Laboratory Animal Center. Isolation and culture of ventricular CMs were performed as previously described, followed by anesthetization of neonatal mice with 2% inhaled isoflurane 11. The complete culture medium for the CMs consisted of 85% Dulbecco's modified Eagle's medium (DMEM)/F-12 (Gibco-BRL, USA), 10% fetal bovine serum (FBS, Gibco-BRL), penicillin (100 U/mL), and streptomycin (100 mg/mL). A mouse macrophage cell line (RAW264.7 cells) was provided by Geneseed Biotech (Guangzhou, China), and the complete culture medium contained 85% DMEM, 10% fetal bovine serum (FBS, Gibco-BRL), penicillin (100 U/mL), and streptomycin (100 mg/mL). All cells were cultivated in humidified air with 5% CO2 at 37 °C.

Following euthanasia, adult CMs were isolated from an 8-week-old mouse as reported previously 12. Adult mouse hearts were quickly removed and mounted on a Langendorff device and subsequently perfused with 50 ml digestion buffer containing 15,000 U of type II collagenase (Roche) and 50 μM CaCl_2_ for 10 minutes. Following a 5-minute perfusion with calcium-free perfusion buffer (113 mM NaCl, 4.7 mM KCl, 0.6 mM KH_2_PO_4_, 0.6 mM Na_2_HPO_4_, 1.2 mM MgSO_4_, 10 mM Na-HEPES, 12 mM NaHCO_3_, 10 mM KHCO_3_, 0.032 mM phenol red, 30 mM taurine, 10 mM BDM), individual CMs were extracted by teasing the hearts into minute pieces and triturating them using a Pasteur pipette. The supernatant was then extracted from the cells by centrifuging them at a low speed. Cells from adult mice were cultured in F12 media supplemented with 10% fetal bovine serum (FBS) on laminin (10 g/ml, Life Technologies, 23017015)-coated culture slides.

### Isolation and culture of cardiac macrophages

After thorough rinsing with PBS, the isolated hearts were enzymatically digested. The pup hearts were digested using a solution of 25% (v/v) trypsin and 1% (w/v) collagenase II, while the adult hearts were digested using a solution of 45% (v/v) trypsin and 100mg/mL collagenase II. Following digestion, the cell suspension was obtained, and the macrophages were separated from the heart using the EasySep™ Mouse F4/80 Positive Selection Kit, following the instructions provided with the kit. Depending on the experimental objectives, the isolated macrophages were cultured using high-glucose DMEM medium supplemented with 10% FBS.

### Culture, viral transfection and cellular optogenetic stimulation of vagal neurons

Based on previous research 13, we selected E21 rat embryos to isolate nodose ganglia from the neck region. The tissues were enzymatically digested using an HBSS solution containing 1mg/ml collagenase II and 5mg/ml trypsin II at 37 °C for 50 minutes. After digestion, the resulting suspension was supplemented with 8mL of sterile Leibovitz's L-15 medium containing 20mg/ml fatty acid-free bovine serum albumin (BSA) and 1% penicillin-streptomycin, and the cells were collected by centrifugation. The cell pellet was gently triturated three times in control culture medium (comprising 20mg/ml non-esterified fatty acid BSA, 200mM L-glutamine, 2% insulin-selenium-transferrin, and 125ug/ml NGF in F12/DMEM medium). The cells were then resuspended at a density of 8000 cells per well in custom-made 24-well cell culture plates and incubated at 35.5 °C with 5% CO_2_.

On the first day after plating, half of the culture medium was replaced with control culture medium containing 2µM Ara-C for 48 hours, aiming to eliminate non-neuronal cells in the culture. On the third day, half of the control culture medium was replaced, and on the fourth day, lantivirus transfection was initiated by introducing LV-CAG-hChR2(H134R)-mCherry at a concentration of MOI=10 into the neuronal culture. After 24 hours, the medium was replaced with complete control culture medium. Subsequently, the cell plates were placed into the cellular optogenetic system and exposed to either 470nm blue light or not.

Finally, neural culture medium from different treatment groups was collected as culture supplements for macrophages.

### Virus injection into the vagus nerve and implantation of the light-emitting diode (LED) device

The virus AAV9-CAG-hChR2(H134R)-mCherry was chosen to transfect the left nodose ganglion (LNG). A comparable construct lacking hChR2 (AAV9-CAG-mCherry) was employed as a control. The virus was obtained from OBio (Shanghai, China). The protocol for viral injection and LED implantation was performed as described in a previous study, with minor modifications 14, 15. A small skin incision was made along the midline of the mouse neck for LNG injection. The LNG was observed and exposed when the vagus nerve was carefully dissected from the carotid sheath to the laryngeal branch. Virus solution (AAV9-CAG-ArchT-GFP: 20 ml, 6.25 x 10^12^ vector genomes/ml; AAV9-CAG-GFP: 20 ml, 5.66 x 10^12^ vector genomes/ml) was injected into the vagus nerve as close to the LNG as possible using the Nanojet II device. Four weeks after virus injection, a skin incision was created in the scapular region, and a vasodilator was utilized to establish a subcutaneous tunnel from this incision to the site of vagus nerve exposure. Then, an LED optical fiber (470 nm, Convergence Technology, Wuhan, China) was routed through the subcutaneous tunnel directly to the left vagus and LNG, extremely close to the virus injection site. Tissue adhesive was used to firmly fix the LED. The outer end of the LED fiber was connected to a monochromatic LED system for illumination (convergent technology). The light output was set at a fixed level (40% duty cycle, 10-Hz period, 20-ms pulse width, and 3 to 5 mW/mm^2^) to limit heat generation while maintaining illumination efficiency. All electrophysiological parameters were evaluated *in vivo* in the presence or absence of LNG illumination. During the procedures, mice were anesthetized with 2% isoflurane for induction and 1% isoflurane for maintenance.

### AChE histochemical staining and epicardial nerve density analysis

All mice underwent surgery to expose the heart while deeply anesthetized with 5% isoflurane and oxygen. Before surgery, 1000 U of heparin was intraperitoneally administered to avoid thrombus formation in the heart. Modifications were made to the previously described method for preparing the heart 16. We transapically infused 2% warm gelatin into the atria and ventricles, after which we chilled the whole heart with cold PBS, which caused solidification and stretching of the relaxed atrial wall. The hearts were removed and placed on a plate containing cold PBS. The pericardium, lungs, and adipose tissue were extracted from the hearts before fixation in a 4% PFA solution for 30 min at 4 °C. The hearts were then washed for 30 min at 4 °C in a wash solution containing PBS, hyaluronidase (0.5 mg/100 ml), and tetraisopropylphosphoramide (0.5 mmol/L, to inhibit pseudocholinesterase) for AChE staining of the whole heart. The hearts were then treated overnight (12 hours) at 4 °C in AChE staining solution. The AChE staining solution consisted of the following components: 60 mmol/L sodium acetate, 2 mmol/L acetylthiocholine iodide, 15 mmol/L sodium citrate, 3 mmol/L CuSO_4_, 0.5 mmol/L K_3_Fe(CN)_6_, and 0.5 mmol/L tetrasodium diisopropylphosphoramide. Then, 1% Triton-X 100 and 0.5 mg/100 ml hyaluronidase were added to the staining solution before use. All the above chemicals are contained in Supplementary Table 2. After staining, the prepared hearts were preserved in 4% PFA until further analysis.

AChE-stained hearts were imaged using a stereomicroscope. For quantitative analysis of epicardial tract density, digital images were captured at the appropriate magnification. Using ImageJ software, morphometric analysis was performed on the same normalized region of each heart. The density of neural distribution was determined after measuring all AChE-stained neural networks in the area.

### Establishment of MI and apical resection model and pathological evaluation

The mouse MI model was generated as previously described by ligating the left anterior descending coronary artery, and the infarct and fibrotic areas were assessed 12. The apical resection (AR) model of neonatal mice was performed as described in a previous study 13. After MI (14 and 28 days), the hearts were sliced into six sections and incubated in 1% TTC for 10 minutes at room temperature (the white area indicates the infarct area; the red area indicates the normal myocardium). Image-Pro Plus 6.0 (Media Cybernetics, Bethesda, MD) was used to digitally quantify the infarct regions. Following the manufacturer's instructions, Masson trichrome staining was employed to examine cardiac fibrosis (the fibrotic region is blue; the normal myocardium is red). For each animal, the area of cardiac fibrosis was determined by evaluating five random images, and the percentage was calculated using ImageJ Analysis software (NIH).

### Pharmacological administration *in vitro* and *in vivo*

For administration of pharmacological agents to macrophages, we mixed LPS (1 µg/ml), IL-4 (2ng/mL), NGF (10ng/mL) and IL-10 antibody (50ug/mL) (Supplementary Table 1) with the corresponding culture medium and cultivated the cells for 48 hours until further procedures. We additionally cultured CMs in the supernatants from the treated macrophages and changed the medium every day to confirm the impacts on the CMs. Depleting macrophages required daily injections of clodronate liposomes (CLD, from Liposoma B.V. Amsterdam) (5 mg/ml) into P1 neonatal mice for 3 days. This dosage was decided upon after consulting both the manufacturer's instructions and the previous scientific literature. The control group received a similar dose of PBS. Mice were sacrificed after one week, and their hearts were harvested for further investigation.

### Establishment of the mouse vagotomy model

Neonatal mice were anesthetized on ice (or adult mice were anesthetized with 1.5% isoflurane). Then, an approximately 5 mm (1 cm for adults) longitudinal incision was made in the neck area sterilized with alcohol 2-3 mm away from the suprasternal fossa. Under an operating microscope, the salivary gland lobes, fascia and adipose tissue were gently separated with ophthalmic forceps until the carotid-jugular bundle was visible. The vagus nerve was exposed by carefully isolating the carotid artery from the jugular vein. According to a previous study 17, an ophthalmic incision of 3-6 mm of the vagus nerve was adequate to achieve complete dissociation with minimal likelihood of reconnection.

### Cardiac function evaluation

Transthoracic echocardiography using a Vevo 2100 high-resolution imaging system (Visual Sonics, ON, Canada) coupled with a 40-MHz transducer was performed to measure the dimensions and function of the heart before and 1, 14 and 30 days after MI. Speckle tracking imaging (STI) was employed to examine the aberrant mobility of the wall at the regional level. Time to peak was calculated from the parasternal long axis view of the left ventricle via STI using B-mode and M-mode images of the left ventricle. We used an 86-mm quadrature coil on a Bruker Biospec 7T/30 system (Bruker Biospin MRI, Billerica, MA) for both transmission and reception of the cardiac MRI (CMR), according to a previous study 18. For each slice, we assessed the angle at which the myocardium showed hyperintensity in the left ventricular wall and divided the angle by 360° to determine the percentage of infarction. We then calculated the percentage of myocardial infarction for each scanned slice.

### Immunofluorescence

Immunofluorescence staining was carried out exactly as reported previously 19. The cells were fixed with acetone, permeabilized with 0.2% Triton X-100, blocked in 3% BSA, and then incubated with primary antibodies against cTnT (1:50, sc-20025; Santa Cruz Biotechnology), Ki67 (1:100, ab15580; Abcam, Cambridge, UK), p-Histone H3 (pH3) (1:100, AF1180; Beyotime, Shanghai, China), Aurora B (1:100, ab2254, Abcam, Cambridge, UK), IB4 (1:100, 217660, Millipore Sigma), CD206 (1:100, 18704-1-AP, proteintech) and COL3A1 (1:100, NB600-594, Novus Biologicals). For the immunofluorescence staining of vagus nerves and LNG, the primary antibodies were anti-beta-III tubulin (1:100, ab78078; Abcam, Cambridge, UK), anti-choline acetyltransferase (ChAT) (1:100, ab18736, Abcam, Cambridge, UK), and anti-NGF (1:100, ab52918, Abcam, Cambridge, UK). hChR2 was a Cy3-labeled autofluorescent protein. After an overnight incubation at 4 °C, the sections were incubated at room temperature with the appropriate secondary antibodies for a period of two hours. The nuclei were stained with DAPI. EdU incorporation was evaluated by utilizing a Click-iT EdU Alexa Fluor 555 Imaging Kit (Invitrogen) according to the manufacturer's instructions. The antibodies used are listed in Supplementary Table 3. The number of positive cells was determined by confocal microscopy (Carl Zeiss, LSM880) of five random fields that were located in inconsecutive sections.

### Quantitative real-time polymerase chain reaction (qRT‒PCR)

qRT‒PCR was carried out in accordance with a previously published method 20. To extract total RNA from CMs, we used an E.Z.N.A.® Total RNA Kit II manufactured by Norcross, GA, USA. mRNA analysis was performed using a LightCycler 480 II System (Roche Diagnostics, Basel, Switzerland) and a SYBR Green PCR Kit (Takara). Supplementary Table 4 contains primer sequences.

### Western blot analysis

Western blot analysis was carried out as previously described, and ImageJ Analysis software (NIH, Bethesda, MD) was used to evaluate protein expression 21. Supplementary Table 5 contains the primary antibodies.

### Flow cytometry

According to a previous study 22, the cardiac tissues were digested in a buffer containing collagenase type I, hyaluronidase, and DNase I at 37°C for 1 h. Single-cell suspensions derived from myocardial tissue were incubated with antibodies against F4/80 (BioLegend, #123110), CD86 (BioLegend, #105006) and CD206 (BioLegend, #141708), which are contained in Supplementary Table 6. All samples were acquired using a BD FACSCantoTM II, and FlowJo10.4 and Modfit 5.0 software were utilized to analyze the data.

### Tube formation and spheroid formation assays

Conditional culture medium of macrophage supernatants was extracted as mentioned above. After preparation, the Matrigel Growth Factor Reduced (BD) Basement Membrane Matrix was incubated for four hours with 1.5 x 10^4^ HUVECs in EBM containing 1% fetal calf serum (FCS) and 20% conditioned medium. Following fixation of the cells with 4% PFA, images of tube formation were acquired using a Zeiss AxioVision microscope (Jena, Germany). To quantify HUVEC spheroids, either the total length of all sprouts on each spheroid or the most significant distance traveled by the migrating cells was used. For each experiment, approximately ten spheroids were examined.

### Measurement of the cytokines

Macrophage culture supernatants or that treated with NGF were collected into tubes, and the cell debris was precipitated by centrifugation to collect the supernatants. The level of soluble cytokines (IL-17A, IL-10, IRF5, Arg1, YM1, IL-4, TGF-β1, EGF, IL-13) in the supernatants was determined by enzyme-linked immunosorbent assay (ELISA) according to the manufacturer's protocols for the Mouse IL17A/IL17 ELISA Kit (KIT51065-1, Sino Biological), Mouse Interleukin 10 (IL10) ELISA Kit (EK14733, SAB), Mouse Interferon regulatory factor 5 (IRF5) ELISA Kit (RK08555, Abclonal), Mouse YM1/Chitinase 3-like 3 ELISA kit (ELM-YM1-1, RayBio), Il4 (Mouse) ELISA Kit (KA3063, Abnova), Mouse TGF-β1 ELISA KIT (EK0506, SAB), Mouse EGF Quantikine ELISA Kit (MEG00, R&D Systems), Il13 (Mouse) ELISA Kit (KA0246, Abnova). Finally, an ELISA plate reader (Spectra Max M5, Molecular Devices, CA, USA) was used to measure the optical density values at a wavelength of 450 nm.

### Statistical analyses

Quantitative data are presented as the mean ± standard deviation and were analyzed using SPSS version 20.0 (SPSS, Inc., Chicago, IL, USA). Student's t test was used for normally distributed datasets when comparing the data from two groups. Differences among multiple groups were analyzed via one-way analysis of variance (ANOVA), followed by the Bonferroni test for data with equal variances and Dunnett's C test for data with unequal variances. Fisher's exact test was performed to analyze aneurysm incidence. A p value < 0.05 indicated statistical significance.

## Results

### The vagus nerve is essential for cardiac repair

**Figure 1:**
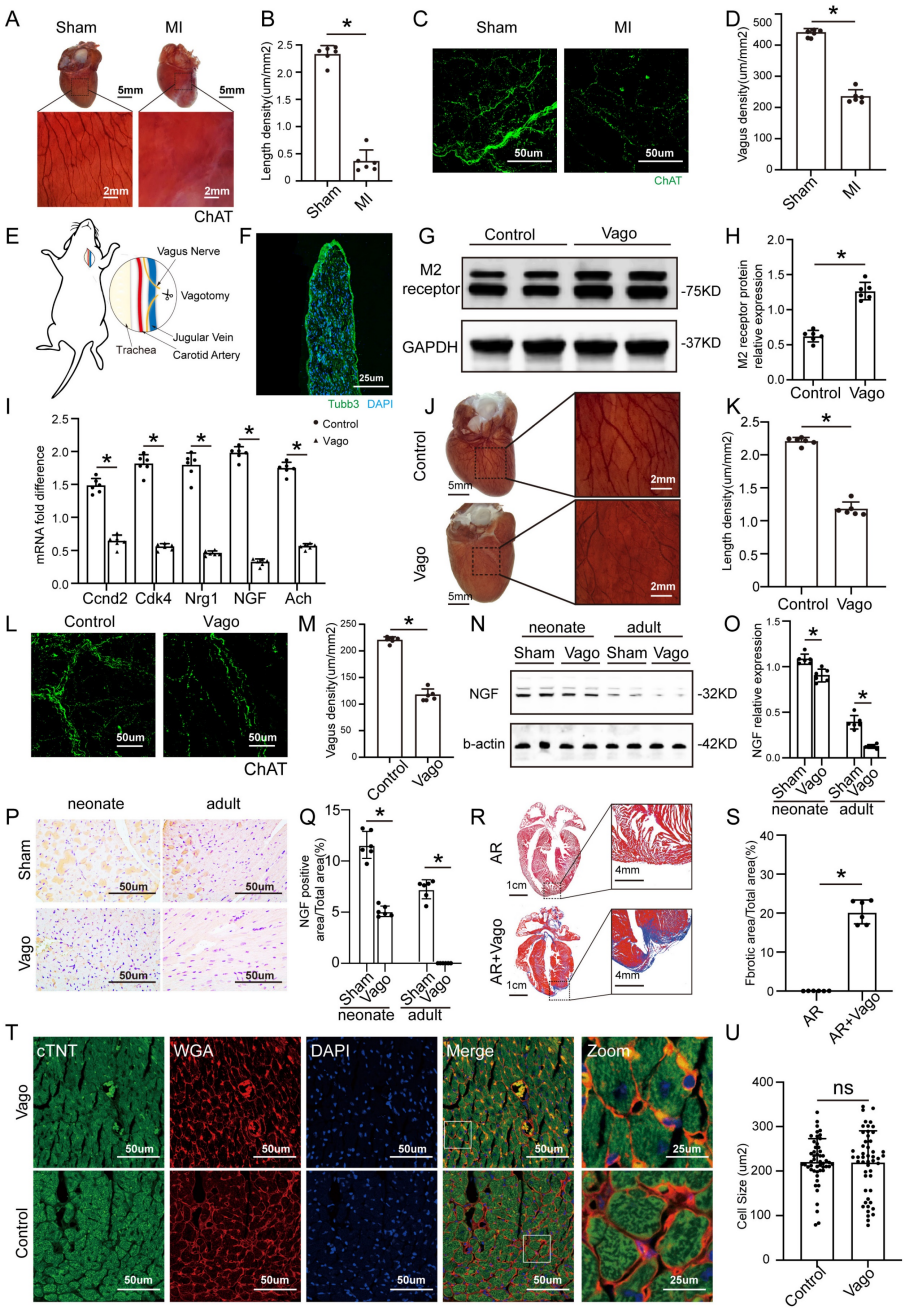
** The vagus nerve was essential for recovery from myocardial injury. (A)** Epicardial nerve bundles stained in the sham and MI groups. **(B)** The length density of epicardial nerve bundles in the sham and MI groups, ⁎p <0.05 vs. sham group; n= 6 per group. **(C)** Representative images of immunofluorescence staining for choline acetyltransferase (ChAT) in the sham and MI groups. **(D)** Quantification of vagal density, ⁎p <0.05 vs. sham group; n= 6 per group. **(E)** Schematic diagram of the procedure used for left cervical vagotomy in mice. **(F)** Immunohistochemistry of the resected vagus nerve stained for tubulin-3 and nuclei stained with DAPI. Scale bar = 25 µm. **(G)** Western blot of the M2 receptor level in control and vagotomized adult mice, showing upregulation of M2 receptor expression following vagotomy. **(H)** Quantification of the M2 receptor data from panel G, ⁎p <0.05 vs. the control group; n= 6 per group. **(I)** qPCR expression profile of Ccnd2, Cdk4, Nrg1, NGF and Ach shows significant downregulation in animals subjected to vagotomy compared to non-operated animals (n = 6, ⁎p < 0.05). **(J)** Photographs show AChE fibers in mice from the control and vagotomy groups. **(K)** Quantification of the length density of epicardial nerve bundles, ⁎p <0.05 vs. control group; n= 6 per group.** (L)** Representative images of immunofluorescence staining for choline acetyltransferase (ChAT) in the control and vagotomy groups. **(M)** Quantification of vagal density, ⁎p <0.05 vs. control group. **(N)** NGF protein levels detected by Western blotting after vagotomy in neonates and adult mice (b-actin was used as the internal reference). **(O)** Quantification of protein levels according to panel N. ⁎p <0.05 vs. sham group. n= 6 per group. **(P)** Immunohistochemistry staining of NGF in the sham and vagotomy groups for neonates and adults. **(Q)** Quantification of the NGF level in the heart according to panel P, ⁎p <0.05 vs. sham group. **(R)** Representative Masson trichrome staining after mouse cardiac apex resection to reveal muscle fibers (red) and collagen (blue) 30 days after vagotomy or sham operation; bars=1 cm and 4 mm, respectively; n= 6 per group. **(S)** Quantitative analysis of the fibrotic area. *P < 0.05 vs. the sham group, n= 6 per group. **(T)** Representative cTnT/DAPI/WGA triple-staining in adult hearts 28 days after surgery. **(U)** Quantitative analysis of cell size, N=6 mice, *P<0.05 vs. the sham group, bar=50 µm. Vago=vagotomy, AR=apex resection.

ChAT histochemistry was performed to label epicardial nerve bundles, and the results showed massive loss of epicardial vagus nerves after MI in adult mice (Figure 1A-B). The same trend was also observed by immunofluorescence (Figure 1C-D). According to a previous study, we constructed a model of left vagus nerve dissection in 1-day-old neonatal mice, and immunofluorescence staining for the pan-neuronal marker beta-tubulin III (Tubb3) confirmed that the nerve was specifically dissected out (Figure 1E-F). Left vagotomy of neonates caused the anticipated upregulation of cholinergic M2 receptor gene expression in ventricular tissue and downregulation of Ach and positive regulators of CM proliferation, such as Ccnd2, Cdk4, Nrg1 and NGF (Figure 1G-I). Furthermore, the density of epicardial vagus nerves was significantly reduced after the left vagus nerve was dissected, as indicated by immunohistochemistry and immunofluorescence for ChAT (Figure 1J-M). NGF, a major factor secreted by the vagus nerve, decreased significantly after vagotomy in neonate and adult mice (Figure 1N-Q). The hearts of neonatal mice had a strong regenerative ability and could be completely regenerated after resection of the apex, but the removed myocardium was merely replaced by fibrotic tissue after vagotomy, as shown by Masson staining (Figure 1R-S). In addition, we evaluated the effect of vagotomy on cardiac hypertrophy, and immunofluorescence staining for WGA showed that vagotomy did not induce cardiac hypertrophy (Figure 1T-U). These data indicated that there is a close relationship between the cardiac vagus nerve and cardiac regeneration.

### Application of optogenetic methods in a mouse model of myocardial infarction

**Figure 2:**
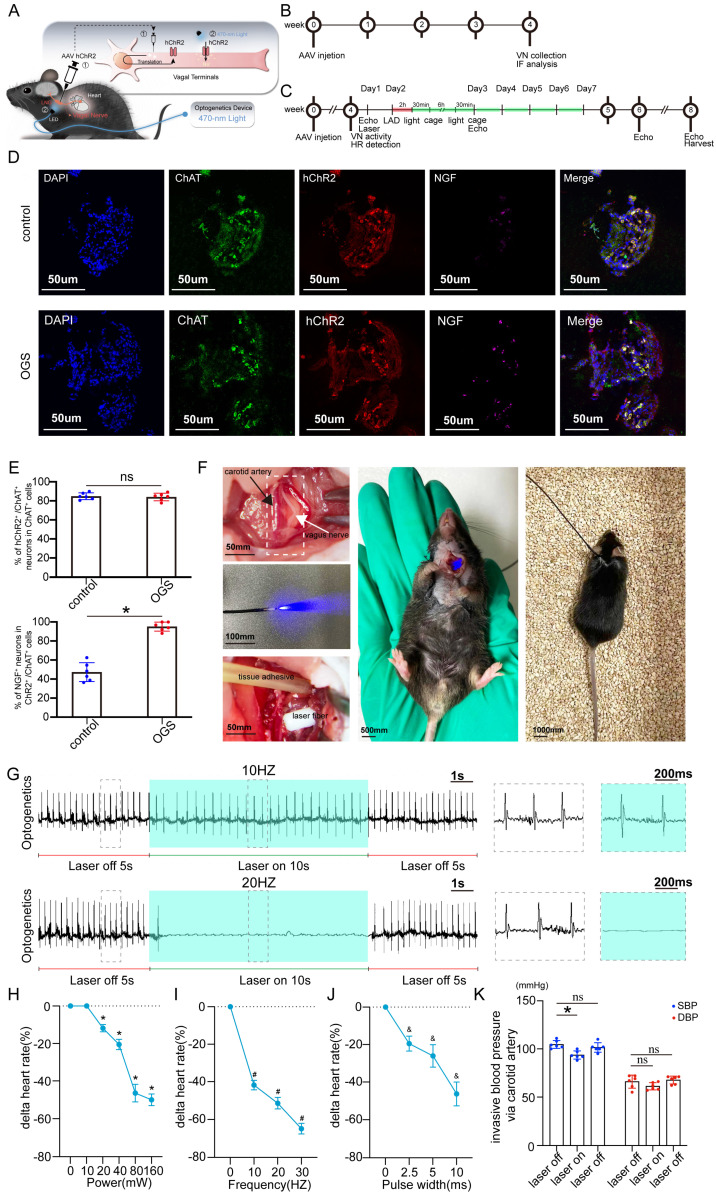
** Construction of an optogenetic model and assessment of the efficacy of vagus nerve stimulation. (A)** Schematic representation of OGS of the left cervical vagus nerve in mice. ① The photosensitive channel protein hChR2 was expressed in the vagus nerve after injection of a virus. ② Light at a wavelength of 470 nm stimulated the vagus nerve via hChR2, which is sensitive to that wavelength of light. **(B-C)** Construction timeline of the adult OGS model. Each time point corresponds to the indicated treatment. **(D)** ChAT/hChR2/NGF triple immunofluorescence staining in the OGS and sham groups. **(E)** Quantitative analysis of the percentage of hChR2-positive cells among neuronal ChAT-positive cells. *P < 0.05 vs. sham group. N=6 mice. **(F)** The implantation procedure for the LED before OGS. The white arrow indicates the vagus nerve, and the black arrow indicates the carotid artery. **(G)** ECG images with varied light frequency stimulation. Bar = 1 s. Enlarged images of the area in the dashed box in (F) at different frequencies are shown. Bar = 200 ms. **(H-J)** The effects of light stimulation at varying energy, frequency, and pulse width values on heart rate variations in mice. *P<0.05 vs. 0 mW; #P<0.05 vs. 0 Hz; &P<0.05 vs. 0 ms. **(K)** Blood pressure variations in mice when the OGS laser was turned off, on, and off again at 10 Hz. *P <0.05 vs. the laser-off group.

The virus solution containing AAV9-CAG-ChR2-GFP was injected into the LNG, and a 2-cm coupled monochromatic light-emitting diode (LED) was inserted and implanted near the LNG (Figure 2A). Four weeks after virus injection, the LNG was harvested for fluorescence staining to evaluate transfection efficiency (Figure 2B). The intervention protocol used after successful viral transfection is shown in Figure 2C. LNG slices were costained with anti-ChAT to label vagal neurons and with anti-ChR2 to label light-sensitive proteins. NGF was used to show vagal activity. The results showed that ChR2 was mainly expressed in ChAT-positive neurons in the LNG in both the optogenetics and control groups; moreover, optogenetics significantly increased vagal activity (Figure 2D-E). Optogenetics were performed under illumination from a light-emitting diode at a wavelength of 470 nm (Figure 2F). We also explored the effect of different light frequencies on heart rate and found that light at 20 Hz can make the heart stop beating; 10 Hz was selected as the frequency parameter for subsequent experiments (Figure 2G). The power, frequency and pulse width values of light were inversely related to the heart rate (Figure 2H-J). A power of 20 mW, frequency of 10 Hz and pulse width of 10 ms were set as the operating parameters. Under the stimulation conditions, systolic blood pressure showed a slight decline, and there was no change in diastolic blood pressure (Figure 2K). These results indicated that our optogenetic approach could achieve cardiac vagal stimulation while hardly affecting the changes in heart rate and blood pressure.

### Optogenetic manipulation promotes CM proliferation, dedifferentiation and angiogenesis after MI

**Figure 3:**
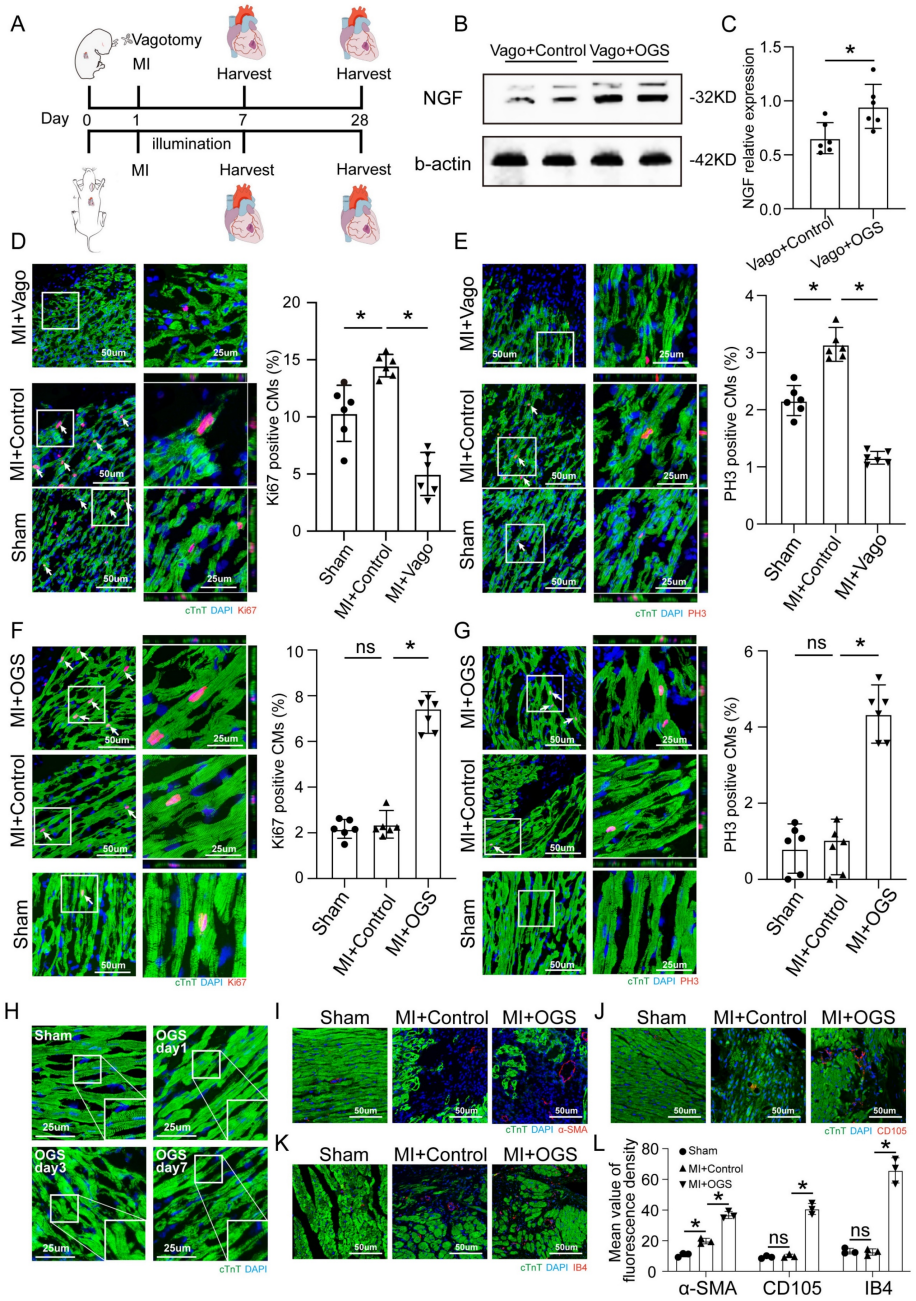
** OGS promoted CM proliferation *in vivo.* (A)** Illustration of the OGS strategy for adult MI mice and the vagotomy treatment for neonatal MI mice. **(B-C)** NGF expression in the neonatal Vago+Control and adultVago+ OGS groups. *P<0.05 vs. the Vago+Control group. **(D)** Ki-67 immunofluorescence staining and quantification of Ki-67-positive CMs in the sham, MI+Control, and MI+Vago groups 7 days after MI in neonatal mice. Ki-67-positive CMs are indicated by arrows. *P<0.05 vs. the MI group. **(E)** pH3 immunofluorescence staining and quantification of pH3-positive CMs in the sham, MI+Control, and MI+Vago groups 7 days after MI in neonatal mice. pH3-positive CMs are indicated by arrows. *P<0.05 vs. the MI group. **(F)** Ki-67 immunofluorescence staining and quantification of Ki-67-positive CMs in the sham, MI+Control, and MI+OGS groups 21 days after MI in adult mice. Ki-67-positive CMs are indicated by arrows. *P<0.05 vs. the MI group. **(G)** pH3 immunofluorescence staining and quantification of pH3-positive CMs in the sham, MI+Control, and MI+OGS groups 21 days after MI in adult mice. pH3-positive CMs are indicated by arrows. *P<0.05 vs. the MI group. **(H)** Changes in the dedifferentiation of the adult mouse myocardium at different times after OGS. **(I-K)** α-SMA, CD105 and IB4 staining of capillaries at 21 days respective groups. **(L)** Quantitative bar chart corresponding to image I-K. n=6; *P<0.05. Vago=vagotomy.

To assess the effect of optogenetics on cardiac repair after MI, immunofluorescence staining was performed to assess myocardial proliferation, dedifferentiation and angiogenesis. Neonatal and adult mice were subjected to vagotomy and optogenetics, respectively, in the context of MI, and myocardial tissue was removed at different time points for further evaluation (Figure 3A).

The level of NGF shown by Western blotting was used as a validation of vagal intervention (Figure 3B-C). The CMs of neonatal mice showed a strong proliferative capacity after MI, but this proliferative capacity decreased significantly after vagus nerve dissection (Figure 3D-E). In contrast, after MI in adult mice, CMs had only a weak proliferative capacity, which was significantly improved after optogenetic stimulation (Figure 3F-G). The CM dedifferentiation process was reflected by tissue morphology and sarcomeric organization on days 1, 3 and 7 after OGS (Figure 3H). We also observed increased angiogenesis during the OGS-induced adult cardiac regeneration process, as indicated by immunofluorescence staining for α-SMA, CD105 and IB4 (Figure 3I-L). Since cardiac fibroblasts also participate in the post-infarction cardiac repair process, we also investigated the impact of vagal nerve activity on cardiac fibroblasts. Immunofluorescence results showed that whether vagotomy or OGS was performed, there was almost no significant change in the proliferative capacity of cardiac fibroblasts in the infarcted heart (Supplemental Figure 1 A-B). Collectively, our findings suggested that OGS facilitated cardiac repair after MI as a result of increased CM proliferation and dedifferentiation accompanied by angiogenesis.

### Optogenetic manipulation attenuates cardiac remodeling and improves cardiac function after MI

**Figure 4:**
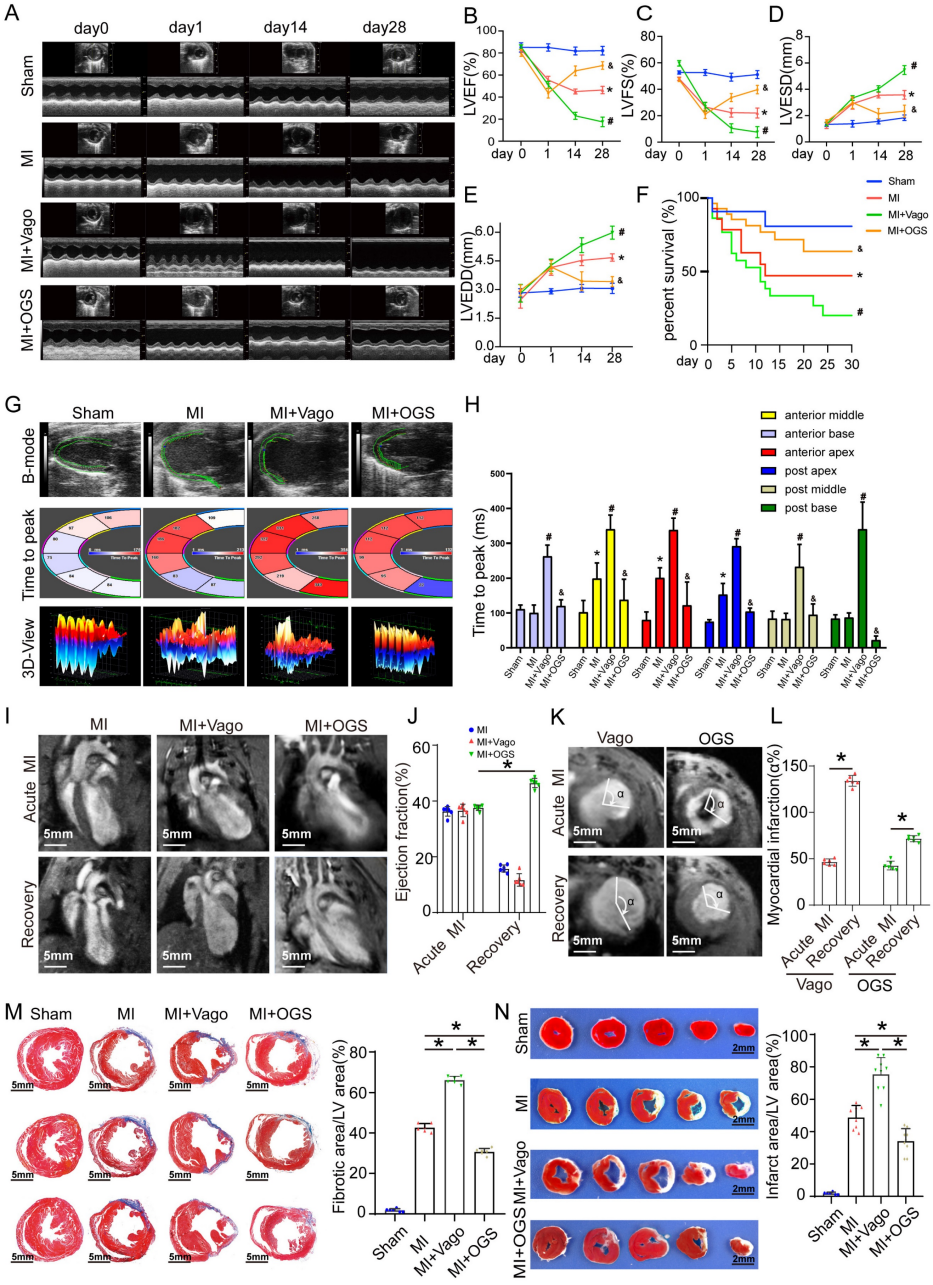
** OGS improved cardiac function after MI. (A)** Cardiac function analyzed by echocardiography on days 0, 1, 14, and 28 after MI. **(B-E)** Corresponding to panel A, quantitative analysis of left ventricular ejection fraction (LVEF), left ventricular fractional shortening (LVFS), left ventricular end-systolic diameter (LVESD), and left ventricular end-diastolic diameter (LVEDD), n=12 per group; *P < 0.05 vs. the sham group on day 28, #P < 0.05 vs. the MI group on day 28, &P < 0.05 vs. the MI+Vago group on day 28. **(F)** Survival curves of each group. **(G-H)** Ultrasound speckle tracking imaging and measurement of left ventricular wall motion in the sham, MI, MI+Vago, and MI+OGS groups. n=12 per group; *P < 0.05 vs. the sham group on day 28, #P < 0.05 vs. the MI group on day 28, &P < 0.05 vs. the MI+Vago group on day 28. **(I-J)** Acute and recovery MRI imaging and EF measurements in various groups. *P < 0.05 vs. acute MI in the MI+OGS group; bar = 5 mm; n=6 per group. **(K-L)** Left ventricular short-axis wall motion on MRI. *P < 0.05 vs. the acute MI group; bar = 5 mm; n=6 in each group. **(M)** Images of Masson-stained cardiac slices 28 days after MI. Serial sectioning was performed at 500 µm intervals, and the fibrotic area was observed in the cardiac slices. *P < 0.05; n = 6 per group; bar = 5 mm. **(N)** Triphenyltetrazolium chloride staining of cross-sections of mouse ventricles 28 days after MI. *P<0.05 (sham, n=6; MI, n=8; MI+Vago, n=9; MI+OGS, n=9); bar = 2 mm.

Effective myocardial regeneration was critical to myocardial recovery after MI. Next, we investigated the effects of optogenetics on cardiac remodeling after MI. From day 14 to 28 after MI, cardiac function reflected by the left ventricular ejection fraction (LVEF), left ventricular fractional shortening (LVFS), left ventricular end-systolic diameter (LVESd), and left ventricular end-diastolic diameter (LVEDd) significantly deteriorated in the vagotomy group, while these parameters were preserved in the optogenetic group (Figure 4A-E). Survival analysis suggested that optogenetics could significantly increase the survival rate after MI (Figure 4F). Moreover, the improved global performance was also reflected by mitigated left ventricular contractility (Figure 4G-H). Two days after MI, the mice in the three groups had similar infarct sizes, as determined by late gadolinium enhancement (LGE) and EF (measured by MRI). Twenty-eight days after MI, mice subjected to optogenetics had an MRI-measured EF of 43%, compared with 18% in control mice and 15% in vagotomy mice (Figure 4I-L). We also performed a pathological evaluation. At 28 days after MI, TTC and Masson trichrome staining showed that the infarct areas (white) and fibrotic scars (blue) were increased in mice treated with vagotomy, and optogenetics significantly reversed these changes (Figure 4M-N). The above results indicated that OGS could obviously improve post-MI cardiac function and prognosis.

### Vagal protection loss leads to pronounced inflammatory macrophage upregulation in cardiac injury

**Figure 5:**
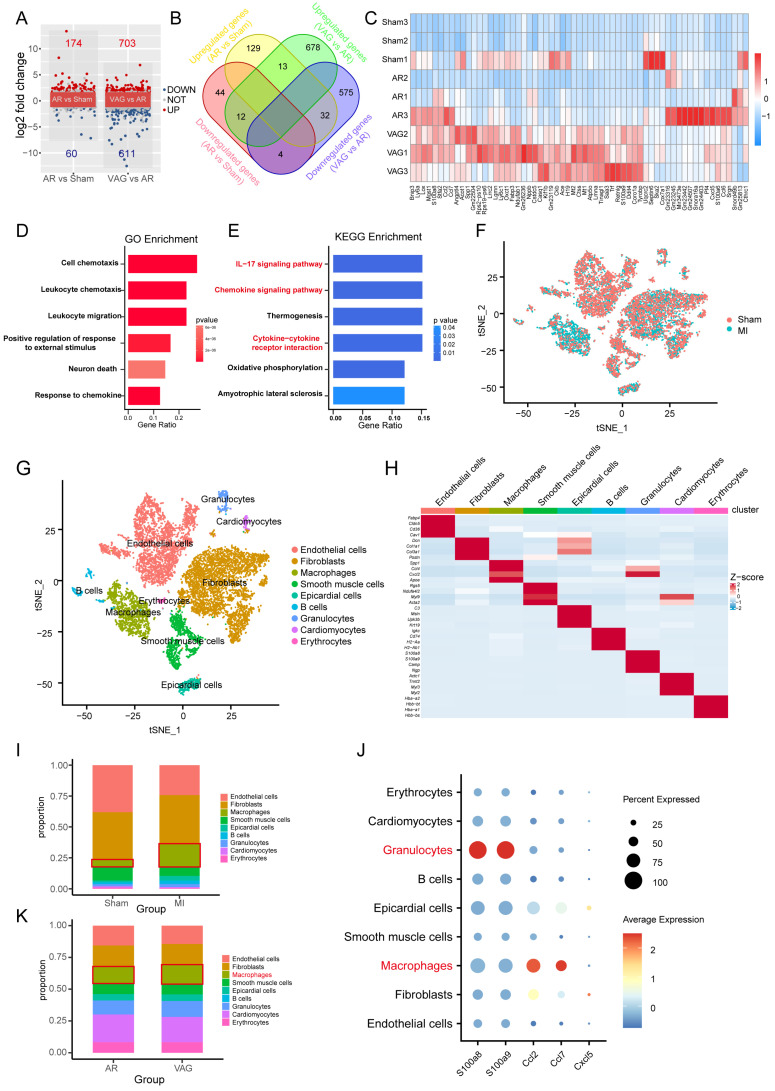
** Vagal protection loss leads to pronounced inflammatory macrophage upregulation in cardiac injury. (A-B)** Volcano and Venn diagrams depicting the differentially expressed genes in the AR vs Sham group and VAG vs AR group. **(C)** Heatmap showing the expression of 61 genes obtained from the intersection of image B. **(D-E)** GO and KEGG enrichment analysis performed on these 61 genes. **(F)** tSNE-based clustering of 3 sham and 2 infarction samples using single-cell transcriptome sequencing data (GSE153481) from a public database. **(G-H)** Categorical annotation of different cell types. **(I)** Changes in the expression ratio of cells in each group. **(J)** Expression of 61 differential genes annotated to 5 closely related genes of the IL-17 signaling pathway, differentially expressed in the AR vs Sham group and VAG vs AR group. **(K)** ssGSEA analysis revealed changes in the proportion of various cell types in the VAG vs AR group, and the results indicate that macrophage changes were predominantly up-regulated among inflammatory cells.

To further investigate the molecular mechanism of vagotomy's effect on post-infarction in mice, we analyzed the transcriptome data (GSE69855) from the public database. We generated volcano and Venn diagrams to illustrate the differentially expressed genes in the AR vs. Sham group and the vagotomy (VAG) vs. AR group (Figure 5A-B). Subsequently, an expression heatmap displayed 61 genes in the intersection set (Figure 5C). These 61 genes were then subjected to GO and KEGG enrichment analysis, indicating that the enriched pathways primarily involved the IL-17 signaling pathway, Chemokine signaling pathway, and cytokine-cytokine receptor interaction (Figure 5D-E). Simultaneously, in order to determine the main affected cell types and the corresponding cell-cell interactions, we integrated single-cell transcriptome sequencing data (GSE153481) from a public database that comprised 3 sham samples and 2 infarction samples. After performing tSNE dimensionality-reducing clustering (Figure 5F), we categorized and annotated the different cell types (Figure 5G-H), and examined the changes in the expression proportions of the cells in each group (Figure 5I). Combining the aforementioned samples, we further investigated the expression of 61 differentially expressed genes within 5 genes closely related with the IL-17 signaling pathway, which were annotated in the single-cell dataset. We observed that their expression was mainly concentrated in granulocytes and macrophages (Figure 5J). Additionally, we analyzed the expression of the top 20 marker genes in each cell type using ssGSEA to assess the changes in the proportions of each cell type in the VAG vs. RES group. The results indicated a predominant up-regulation of macrophages among the inflammatory cells (Figure 5K).

### Vagus nerve activation promotes M2-type polarization of macrophages

**Figure 6:**
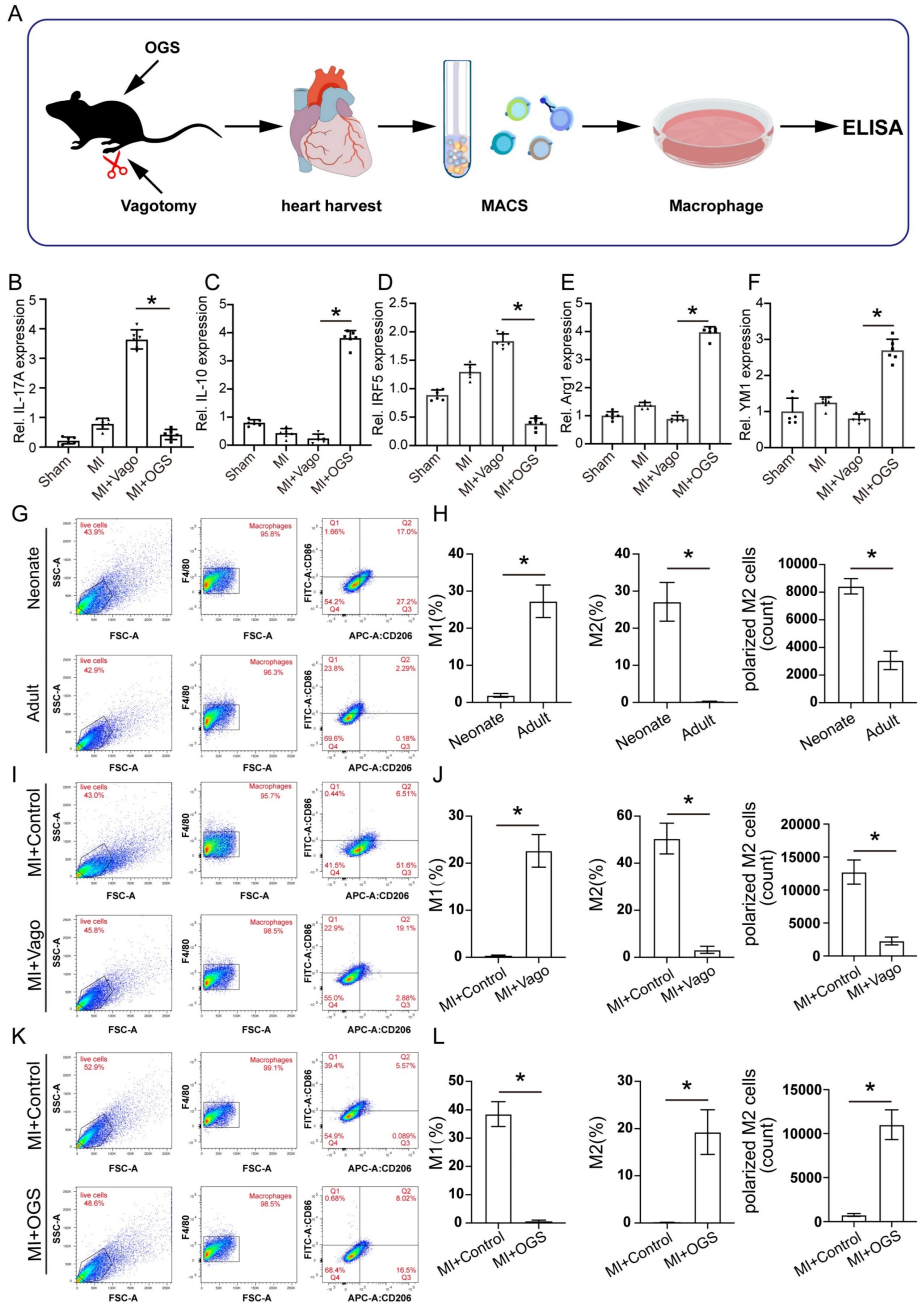
** Vagus nerve activation promotes M2-type polarization of macrophages. (A)** Experimental flowchart. Macrophages from 3 to 5 isolated hearts were obtained for each cell culture plate. **(B-F)** Concentrations of certain several cytokines in macrophage culture media from different treatment groups. *P < 0.05; n = 6 per group. **(G-H)** Representative flow cytometry images of cardiac macrophages in neonatal and adult mice, along with quantitative analysis of M1 and M2 polarization ratios. *P < 0.05; n = 6 per group. **(I-J)** Representative flow cytometry images and quantification of M1 and M2 polarization rates in cardiac macrophages of neonatal mice from the MI+Control group and MI+Vago group. *P < 0.05; n = 6 per group. **(K-L)** Representative flow cytometry images and quantification of M1 and M2 polarization rates in cardiac macrophages of aged mice from the MI+Control group and MI+OGS group. *P < 0.05; n = 6 per group.

To further investigate the specific regulatory role of the vagus nerve on cardiac macrophages, we collected hearts from mice that underwent either vagotomy or OGS therapy following MI. Subsequently, we utilized magnetic-activated cell sorting (MACS) to isolate macrophages (Figure 6 A) from the cardiac tissue for cell culture. Based on the above findings (Figure 5E) indicating a correlation between gene expression and the IL-17 signaling pathway after vagus nerve loss, we employed enzyme-linked immunosorbent assay (ELISA) to detect the levels of several soluble cytokines in the cultured macrophage medium, evaluating the inflammatory state of macrophages in infarcted hearts following vagotomy or OGS. The results revealed that vagotomy further increased the levels of IL-17A but decreased IL-10 levels in infarcted hearts, whereas OGS demonstrated the opposite effect (Figure 6 B-C). Impressively, OGS intervention led to a reduction in IRF5 expression, representative of M1 macrophages, while elevating the expression of Arg1 and YM1, representative of M2 macrophages. These findings suggest a distinct inhibitory effect on M1 macrophages and a promotion of M2 polarization (Figure 6 D-E). Furthermore, flow cytometry analysis of cardiac macrophages demonstrated a higher proportion of M2 macrophages and a lower proportion of M1 macrophages in neonatal mouse hearts, which exhibit significant regenerative and reparative capacities, compared to adult mouse hearts that lack such abilities (Figure 6 G-H). Interestingly, vagotomy substantially reversed the predominant ratio of M2 macrophages in neonatal mouse hearts (Figure 6 I-J). Conversely, OGS mitigated the prevalence of M1 macrophages and increased the proportion of M2 macrophages within adult mouse hearts (Figure 6 K-L). Similarly, immunofluorescent staining of M2 macrophages in the sliced heart tissue also showed comparable results (Supplementary Figure 2 A-B). These compelling results collectively suggest that augmented vagal activity can induce polarization of cardiac macrophages towards the M2 phenotype.

### Activated vagual nerve-induced M2 macrophage polarization promotes CM proliferation and endothelial cell activation

**Figure 7:**
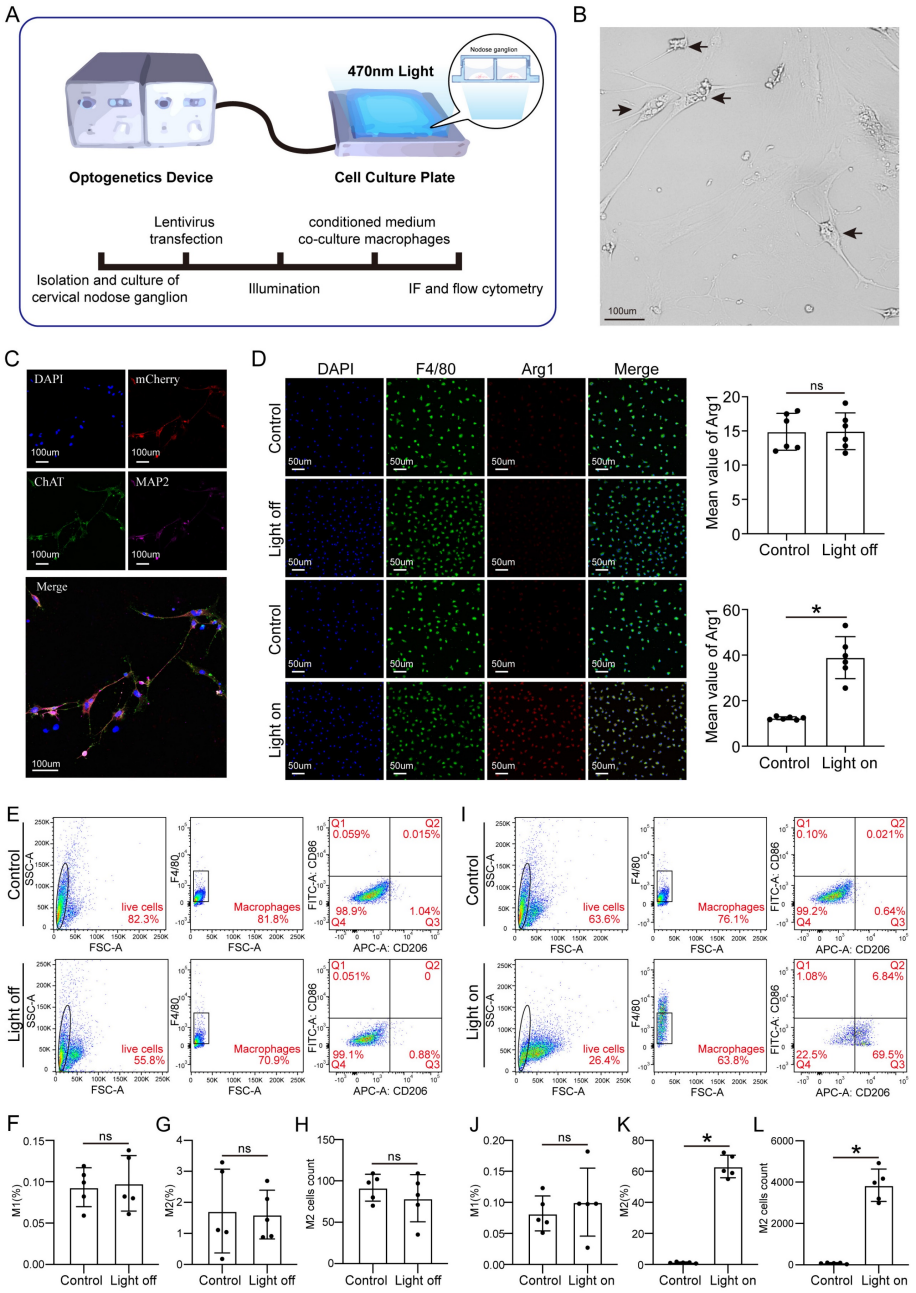
** Cellular optogenetic stiumatlion of vagual neurons induces M2 macrophage polarization. (A)** A schematic of cellular optogenetic stimulation of vagual neurons *in vitro* and the experimental flowchart. **(B)** Bright field image of isolated and cultured vagus neurons. Arrows indicate neuronal cytosol. **(C)** Immunofluorescence labeling of vagal neurons following lentiviral transfection. DAPI represents the nuclei, mCherry represents transfected viral fluorescence, CHAT represents vagus nerve markers, and MAP2 represents neuronal markers. **(D)** Immunofluorescence labeling of Arg1 in cultured macrophages from neuronal medium under light-on or light-off conditions, assessing the impact of vagal stimulation on M2 macrophage polarization. *P < 0.05; n = 6 per group **(E)** Flow cytometry analysis of M1 macrophage polarization in cultured macrophages from neuronal culture medium under light-on or light-off conditions. **(F-H)** Proportion of M1 and M2 macrophages and the number of M2 macrophages corresponding to the image E. *P < 0.05; n = 5 per group **(I)** Flow cytometry analysis of M2 macrophage polarization in cultured macrophages from neuronal medium under light-on or light-off conditions. **(J-L)** Proportion of M1 and M2 macrophages and the number of M2 macrophages corresponding to the image I. *P < 0.05; n = 5 per group

To further investigate how vagal neurons influence CMs through macrophages, we conducted *in vitro* experiments following the schematic diagram (Figure 7A). Firstly, we isolated and cultured vagal neurons from the cervical nodose ganglia of E21 rat embryos and transfected them with LV-CAG-hChR2, and confirmed the transfection efficiency (Figures 7B-C). Subsequently, we treated the macrophages with the medium from the cultured vagal neurons with or without OGS. We found that conditioned medium from neurons stimulated with OGS increased the expression of Arg1, a marker of M2 macrophages. However, in the absence of specific wavelength light stimulation, the transfected hChR2 remained inactive, thus failing to polarize the macrophages (Figure 7D). Flow cytometry results also showed that conditioned mediums from light-stimulated neurons promoted macrophage polarization towards the M2 phenotype (Figures 7E-L).

To further validate the effect of polarized macrophages on CM proliferation, we added different macrophage-conditioned mediums to CMs. First, we induced M0 macrophages into M1 or M2 polarization by adding LPS or IL-4 respectively (Supplementary Figure 3 A). Flow cytometry confirmed the successful polarization induction (Supplementary Figure 3 B-C). To verify the effects of the macrophage polarization agents themselves, we directly added LPS or IL-4 to cultured CMs and evaluated their proliferative capacity. The results showed that LPS inhibited CM proliferation (Supplementary Figure 4 A-D), while IL-4 did not enhance the ability (Supplementary Figure 4 E-H).

Next, we treated 1-day-old and 7-day-old mouse CMs with the conditioned media from M1 and M2 macrophages, after confirming the nearly non-residual LPS and IL-4 in the media (results not shown). The immunofluorescence results showed that M1 macrophage-conditioned media decreased the proportion of CMs positive for Edu, pH3, Aurora B, and Ki67, whereas M2 macrophage-conditioned media increased their proportion (Supplementary Figure 2D-I and Supplementary Figure 5 A-B). Similar results were observed in cultured adult mouse CMs (Supplementary Figure 5 C-E). Moreover, M2 macrophage-conditioned media enhanced DNA synthesis in 7-day-old mouse CMs, as indicated by a higher proportion of cells in the S phase (Supplementary Figure 5 F-H). Taken together, these results suggest that M2 macrophage polarization enhances CM proliferation.

Additionally, we assessed the impact of macrophage polarization on endothelial cells. Endothelial cells treated with M2 macrophage-conditioned media exhibited significant vessel sprouting and tube formation, which is crucial for angiogenesis, whereas M1 macrophages had the opposite effect (Supplementary Figure 5 I-K). These *in vitro* data indicate that optogenetically-induced M2 macrophage polarization promotes cardiomyocyte proliferation and angiogenesis in endothelial cells.

### OGS activates IL-10/STAT3 signaling pathway to promote M2 mcarophage polarization via which itself enhances cardiac regeneration

**Figure 8:**
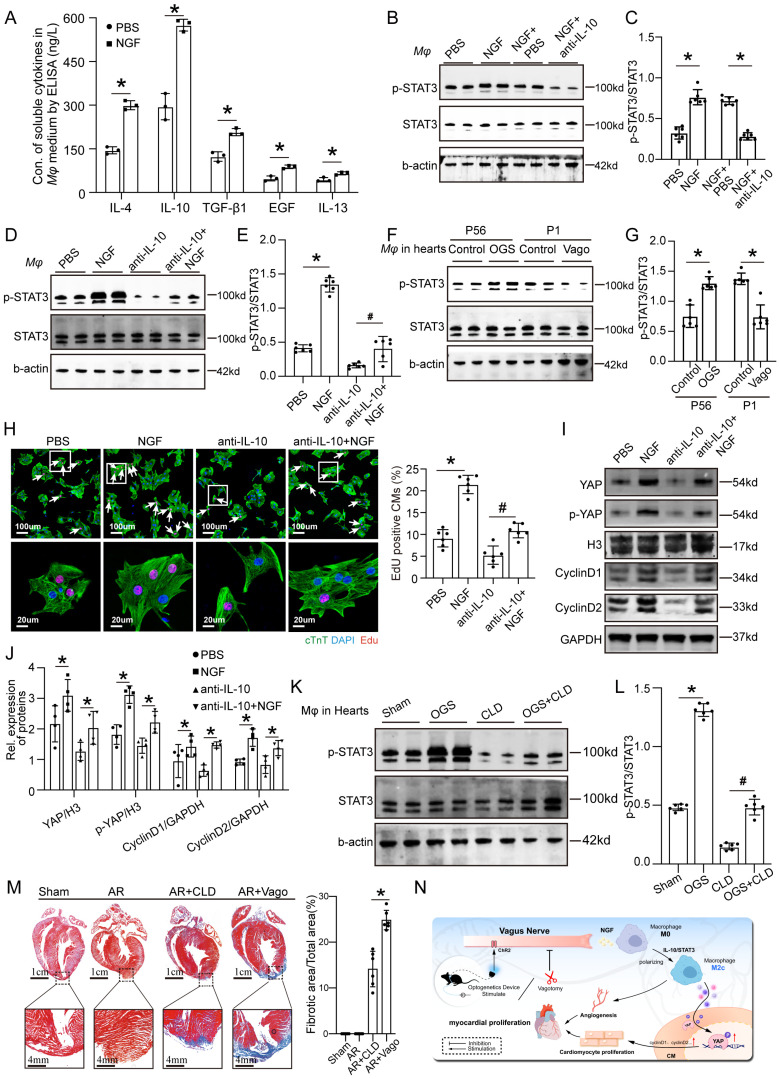
** OGS activates IL-10/STAT3 signaling pathway to promote M2 mcarophage polarization via which enhancing cardiac regeneration. (A)** Concentrations of soluble cytokines in macrophage culture media from the NGF-treated and control groups. *P < 0.05; n = 3 per group.** (B-E)** Western blot detection of p-STAT3 and STAT3 protein levels in macrophages, along with corresponding statistical analysis results, after different agents aderministration in macrophages. *P < 0.05; n = 6 per group. **(F-G)** Protein expression levels of p-STAT3 and STAT3 in macrophages from corresponding cardiac tissues after the administration of GOS in infarcted adult mice, as well as after vagotomy in infarcted neonatal ones, and the results of their statistical analysis. *P < 0.05; n = 6 per group. **(H)** Immunofluorescence labeling with EdU and cTnT in P7 CMs cultured in macrophage-conditioned cultures treated with PBS, NGF, anti-IL-10, and anti-IL-10+NGF. White arrowheads indicate EdU-positive CMs. *P < 0.05; n = 6 per group. **(I-J)** Western blotting and quantitative analysis of YAP, p-YAP, CyclinD1, and CyclinD2 protein levels in P7 CMs cultured with condition medium from macrophages treated with PBS, NGF, anti-IL-10, and anti-IL-10+NGF. *P < 0.05; n = 4 per group. **(K-L)** Western blotting and quantitative analysis of p-STAT3 and STAT3 protein levels in macrophages from different adult mice groups (Sham, OGS, CLD, and OGS+CLD). *P < 0.05; n = 6 per group. **(M)** Representative Masson staining of the Sham, AR, AR+CLD, and AR+Vago groups showing myofibrils (red) and collagen (blue). AR=apex resection, Vago = vagotomy. *P < 0.05; n = 6 per group. **(N)** Illustration of the mechanism by which OGS regulates CM proliferation and angiogenesis through macrophage polarization.

As mentioned earlier, NGF exhibited increased expression and secretion after OGS, suggesting that OGS-induced M2 macrophage polarization may be mediated by NGF. To test this hypothesis, we measured the concentrations of soluble cytokines in the medium of macrophages after NGF treatment. The results showed significantly higher levels of several cytokines associated with M2 macrophages compared to the control group, with IL-10 showing the most prominent change (Figure 8A). Previous studies have shown that the IL-10/STAT3 signaling pathway promotes M2 macrophage polarization 23, and M2 macrophages themselves secrete IL-10 24, forming a positive feedback loop that further enhances M2 polarization. Western blot analysis demonstrated that NGF-treated macrophages expressed higher levels of phosphorylated STAT3 compared to the negative control group, and this elevation was weakened by the application of an IL-10 antibody (Figures 8B-C). Conversely, NGF treatment rescued the inhibitory effect of the IL-10 antibody on STAT3 phosphorylation (Figures 8D-E). Further investigation revealed that the phosphorylation levels of STAT3 in macrophages in the hearts of adult mice increased after OGS, while neonatal mice with vagotomy had lower levels of STAT3 phosphorylation in their hearts (Figures 8F-G). We also found that the conditioned medium from NGF-treated macrophages significantly increased the number of Edu-positive CMs from 7-day-old mice, while the conditioned medium from IL-10 antibody-treated macrophages suppressed CM proliferation, and NGF partially reversed this inhibitory effect (Figure 8H). YAP protein in CMs is known to be closely related to their proliferative capacity 25. Therefore, we further analyzed how NGF-induced M2 macrophage polarization regulates CM proliferation. Western blot analysis of proteins from CMs subjected to different treatments revealed that the conditioned medium from NGF-treated macrophages increased the expression of YAP, p-YAP, cyclinD1, and cyclinD2 in CMs, while the application of an IL-10 antibody resulted in the opposite trend, and NGF counteracted the detrimental effect of IL-10 antibody (Figure 8I-J). These results indicate that M2 macrophage polarization requires NGF-induced activation of the IL-10/STAT3 signaling pathway and may enhance CM proliferation through the secretion of cytokines that increase the expression of YAP protein. CLD was used to deplete macrophages *in vivo*. The p-STAT3 levels in macrophages in the hearts of mice significantly decreased after treatment with CLD, while OGS increased the expression of p-STAT3 in residual macrophages (Figures 8K-L). We also observed that macrophage depletion and vagotomy led to impaired regenerative repair in a neonatal AR mouse model (Figure 8M). These results indicate that macrophage is essential for cardiac regeneration after injury. In summary, our findings suggest that the vagal cholinergic signaling pathway may induce M2 macrophage polarization through the secretion of NGF, which activates the IL-10/STAT3 pathway. M2 macrophages, in turn, enhance CM proliferation through a paracrine mechanism involving the YAP signaling (Figure 8N).

## Discussion

In this study, we confirmed the pivotal role of cardiac vagal stimulation in myocardial regeneration and functional repair after MI. Optogenetics that were used to activate the cardiac vagus nerve could significantly facilitate CM proliferation and angiogenesis, thus improving cardiac function after MI in adult mice. In terms of mechanism, cardiac vagal stimulation initiated the IL-10/STAT3 signaling pathway and induced polarization of cardiac macrophages toward the M2 type, resulting in myocardial regenerative repair. Overall, we have demonstrated that targeted cardiac vagal stimulation is capable of promoting cardiac regeneration, which may provide new opportunities for the clinical treatment of HF.

Our study further reinforced the necessity of cardiac vagal function in myocardial regeneration, and crucially, we clarified that cardiac vagal modulation could promote postnatal myocardial regeneration and cardiac function improvement. Increasing evidence has shown that parasympathetic nerves are required to guide CM proliferation 5, 26. Consistent with these findings, in our study, ChAT staining showed that MI caused a significant decrease in vagus nerve density and that vagotomy led to scar repair after AR in neonatal rats. More importantly, in the current study, our findings confirmed that cardiac vagal stimulation could promote myocardial regeneration and improve post-MI cardiac function, which not only clarified the important role of vagal intervention in myocardial regeneration but also provided a new strategy for the treatment of HF.

A recent study reported that cardiac vagal activation could promote myocardial repair after MI 27, but whether this beneficial effect was induced by cardiac regeneration had not been declared clearly. Nerve-regulated regenerative responses in many tissues appeared to be an evolutionarily conserved pathway among different species 28. In the present study, we showed that vagal stimulation allowed mature myocardium to dedifferentiate, a marker that myocardium had regained its ability to proliferate 29. Moreover, we evaluated the CM proliferative effect, angiogenesis and improvement in post-MI cardiac function, which showed that vagal stimulation significantly increased Ki-67- and pH3-positive CMs, promoted angiogenesis and then achieved cardiac functional repair upon damage. Taken together, our results showed that cardiac vagal stimulation was indispensable for CM proliferation and further suggested that cardiac vagal stimulation could promote myocardial regeneration, thus effectively improving post-MI cardiac function.

The strategy of neural activation by applying molecular targets was limited by the weak efficacy of single target, lack of physiological activation properties, and insufficient transfection efficiency. Compared with these, optogenetics, a method that was used to selectively stimulate the cardiac vagus nerve, could be an ideal approach to promote myocardial regeneration after MI. The internal cervical vagus nerve contains many branches, and the LNG was more likely to project to the cardiac vagus nerve due to its proximity to the cardiac vagus ganglion 30. As required, we transduced a specific light-sensitive channel into the LNG; then, by illuminating the site with a light-emitting diode (470 nm) 31, we selectively activated the cardiac vagus nerve. The ChR2 gene that had been used in CM depolarization and contraction was the core medium to mediate targeted neural intervention in the optogenetic procedure 32, 33. Similarly, our current study used adeno-associated viral vectors to deliver ChR2 into the LNG and selectively activate the cardiac vagus nerve, which was of great value in promoting myocardial regenerative repair. Previously, electrical current pulses for the cervical vagus nerve were used to activate the cardiac vagus nerve to treat HF, but the effect was limited because this electrical stimulation could not distinguish among the internal cervical vagal tract and failed to target the cardiac vagus nerve 34, 35. The application of an optogenetic approach to control different types of cardiac cells for cardiac pacing, defibrillation or cardioversion, and arrhythmia termination indicated its ability to selectively stimulate target neurons 36. In the current study, we verified that optogenetics could realize cardiac vagus nerve-targeted activation, therefore promoting myocardial regeneration and improving myocardial repair after injury. The above results expanded the application of optogenetics in the field of myocardial regeneration, indicating that optogenetics could be an effective method to improve myocardial regenerative repair by selectively stimulating the cardiac vagus nerve. In addition, we evaluated the effect of optogenetics on heart rate and blood pressure, which showed that the current parameters slightly slowed heart rate and had little effect on blood pressure, demonstrating the safety of this approach. Thus, our data suggested that optogenetics could be an effective and safe means to stimulate the cardiac vagus nerve, thus achieving cardiac regenerative repair after injury.

We further investigated the mechanisms by which the vagus nerve facilitated myocardial regenerative repair and found that cardiac vagal activation increased the level of IL-10/STAT3 signaling, then achieved therapeutic modulation of monocytes/macrophages, the most important agent in the regenerative immune microenvironment 37. Our results revealed that the vagus-macrophage-inflammation axis promoted post-MI myocardial regeneration and improved the prognosis. The specific mechanism of nerve involvement in myocardial regeneration and repair has not been clarified clearly. In the present study, we analyzed previous sequencing data and single cell data after vagal intervention and MI, further observed major changes in inflammation and macrophage. Regenerated macrophages have a unique polarization phenotype and secrete numerous soluble factors that may facilitate the formation of new myocardium 38. After depletion of macrophages in neonatal mice, myocardial repair was mainly accomplished by fibrous scars, similar to the outcome after vagotomy. Interestingly, we found that direct intervention with LPS or IL-4 in CMs had no effect on their proliferation, while IL-4 or optogenetic stimulation increased the proportion of M2-type macrophages, which in turn promoted CM proliferation, including DNA synthesis and the appearance of cytokinesis features, which again demonstrated the promoting effect of M2 macrophages on myocardial regeneration. IL-10 was found to be a key mediator in terms of the nerve-macrophage link by analyzing the sequencing data after vagal intervention. It has been suggested that IL-10 was the inducer of M2-type macrophages polarization and could effectively promote cardiac repair after MI 39. Our cluster analysis results showed that the level of IL-10 and markers for M2 macrophages were significantly decreased after vagotomy. STAT3 phosphorylation via IL-10 signaling has been reported to promote M2-type macrophages polarization and CM survival after myocardial injury 40, 41. In the present study, our data showed that NGF could increase the phosphorylated STAT3 level and rescue the inhibitory effect of anti-IL-10 on the phosphorylation of STAT3. Collectively, our results indicated that cardiac vagal activation mainly induced the polarization of M2 macrophages by activated the IL-10/STAT3 pathway to promote myocardial regenerative repair.

Our study still has some limitations. First, the current study did not specifically analyze the influence of light frequency and time on vagal activation, and further research was needed to explore it in more detail. Then, due to the lack of suitable database, our results for sequencing analysis and single-cell data came from two different databases, which may result in specimen differences, and future analysis using the same database would be more accurate. Finally, our study built a model of acute myocardial ischemia, and investigations of the role of optogenetic strategies in chronic myocardial ischemia are also needed.

## Conclusion

Our data showed that selective activation of the cardiac vagus nerve by OGS could activate the IL-10/STAT3 pathway, thereby inducing the polarization of cardiac M2 macrophages to promote myocardial regeneration and improve cardiac function after MI. These findings suggested that targeted neuromodulation may be an ideal strategy to improve the efficacy of myocardial regeneration.

## Supplementary Material

Supplementary figures and tables.
