# Supplementary material for: Optogenetic Stimulation of the Cardiac Vagus Nerve to Promote Heart Regenerative Repair after Myocardial Infarction
Source: Int J Biol Sci. 2024 Mar 17;20(6):2072–91. doi: 10.7150/ijbs.89883 (PMC11008277; doi:10.7150/ijbs.89883)
Supplement: Supplementary file 1 — Supplementary figures and tables. [file ijbsv20p2072s1.pdf]

Supplementary material

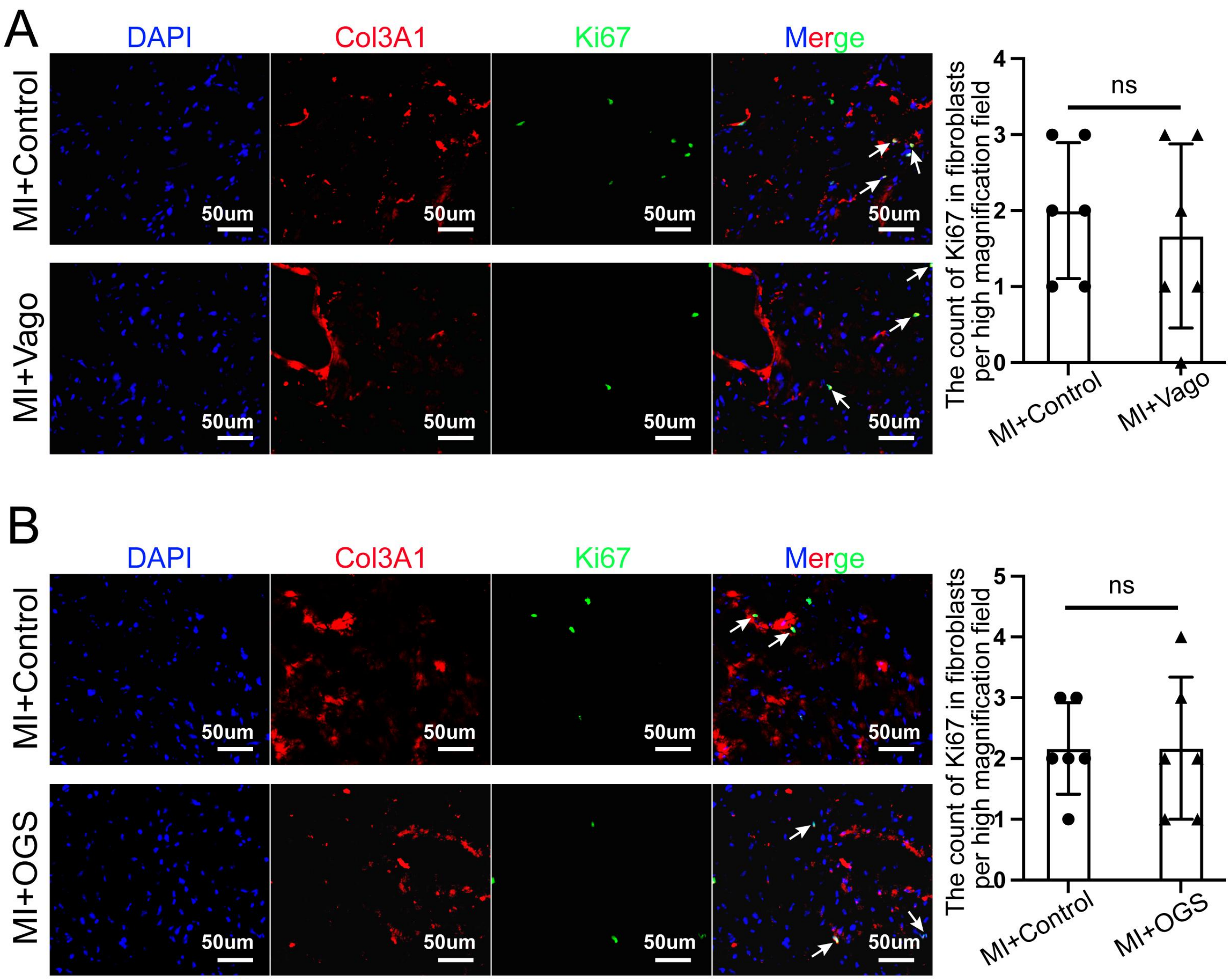
A
DAPI
Col3A1
Ki67
Merge
MI+Control
MI+Vago
50um
The count of Ki67 in fibroblasts per high magnification field
ns
MI+Control
MI+Vago
B
DAPI
Col3A1
Ki67
Merge
MI+Control
MI+OGS
50um
The count of Ki67 in fibroblasts per high magnification field
ns
MI+Control
MI+OGS

2

Supplemental Figure 1 The impact of vagal nerve activity on cardiac fibroblast proliferation

(A) Immunofluorescence staining and quantitative analysis of Ki67 in cardiac fibroblasts in the hearts of neonatal mice 7 days after MI in the MI+Control group and the MI+Vagus group. *P<0.05 vs. MI+Control group. (B) Immunofluorescence staining and quantitative analysis of Ki67 in cardiac fibroblasts in the hearts of adult mice 21 days after MI in the MI+Control group and the MI+OGS group. *P<0.05 vs. MI+Control group. Col3A1= Collagen III alpha 1, represent cardiac fibroblasts.; Vago=vagotomy; OGS= optogenetic stiumatlion.

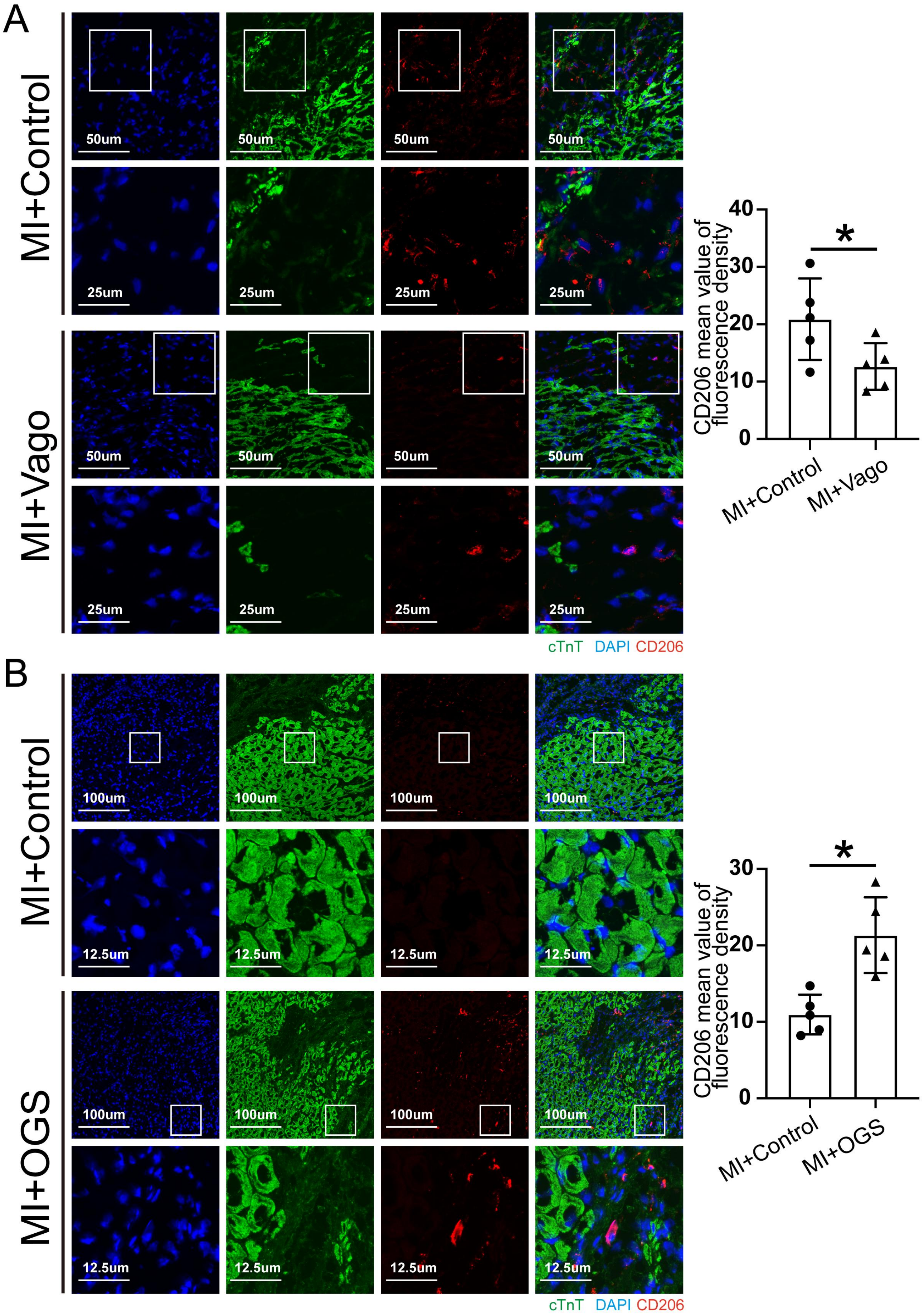
A
MI+Control
MI+Vago
50um
25um
CD206 mean value of fluorescence density
40
30
20
10
0
*
MI+Control
MI+Vago
cTnT DAPI CD206
B
MI+Control
MI+OGS
100um
12.5um
CD206 mean value of fluorescence density
30
20
10
0
*
MI+Control
MI+OGS
cTnT DAPI CD206

25

Supplemental Figure 2 The effect of vagal nerve activity on M2 polarization of cardiac macrophages

(A) Immunofluorescence staining and quantitative analysis of CD206 in cardiac macrophages in neonatal mice 7 days after MI in the MI+Control group and the MI+Vago group. *P<0.05 vs. the MI+Control group. (B) Immunofluorescence staining and quantitative analysis of CD206 in cardiac macrophages in adult mice 21 days after MI in the MI+Control group and the MI+OGS group. *P<0.05 vs. the MI+Control group. Vago=vagotomy; OGS= optogenetic stiumatlion.

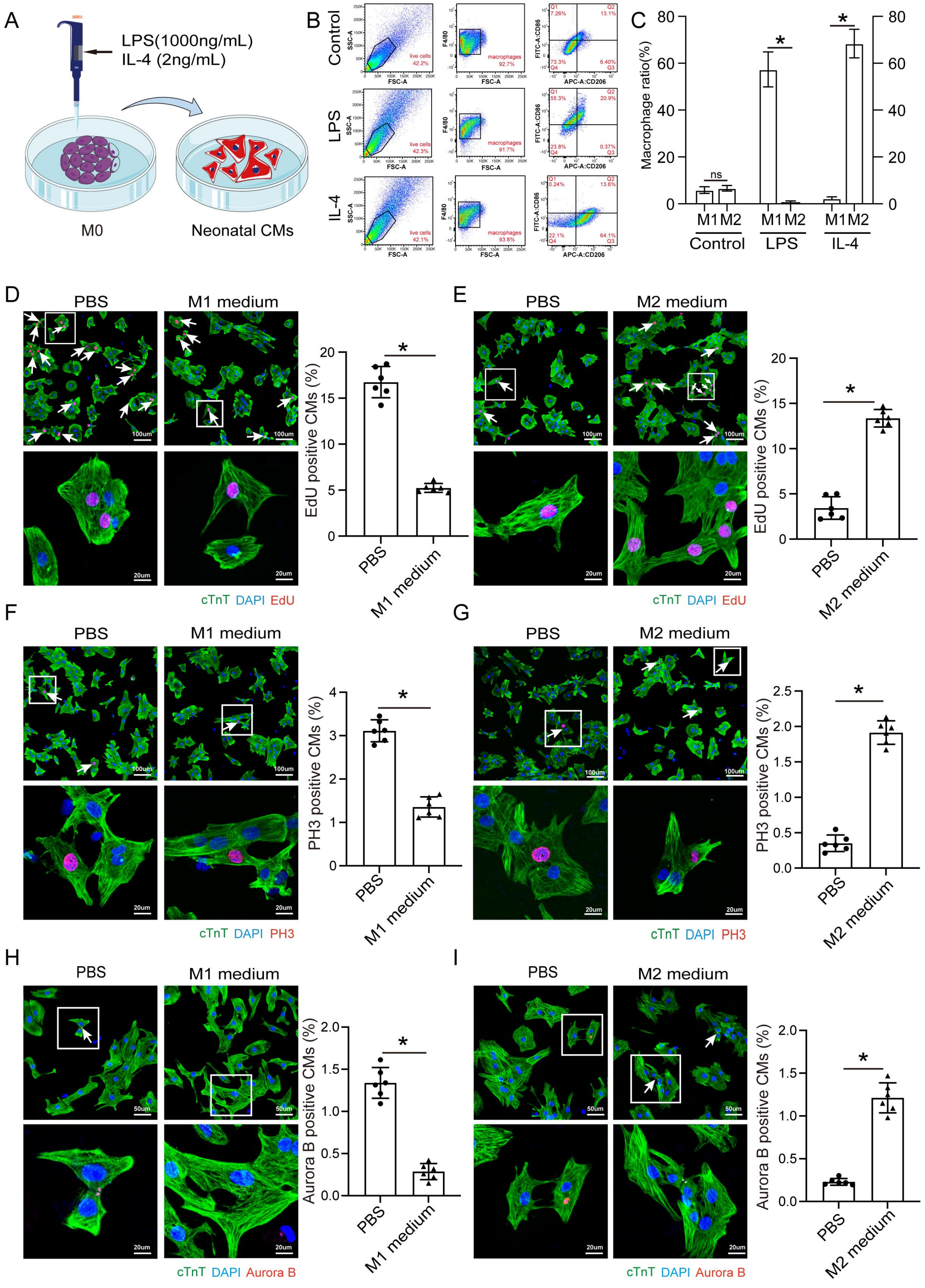

A
LPS(1000ng/mL)
IL-4 (2ng/mL)
M0
Neonatal CMs
B
Control
LPS
IL-4
live cells
macrophages
C
Macrophage ratio(%)
ns
M1 M2
Control
LPS
IL-4
D
PBS
M1 medium
EdU positive CMs (%)
cTnT DAPI EdU
E
M2 medium
F
PH3 positive CMs (%)
cTnT DAPI PH3
G
H
Aurora B positive CMs (%)
cTnT DAPI Aurora B
I

34

Supplemental Figure 3 M2 macrophages induce CM proliferation *in vitro*

(A) Schematic illustration of the activating processing of M0 macrophages and neonatal CMs. (B-C) Flow cytometry and the statistical results showing the effects LPS and IL-4 on macrophage polarization, respectively. *P < 0.05 vs. the M1 group; n = 5 per group. (D-E) Immunofluorescence staining with Edu of P1 and P7 CMs cultured in M1 macrophage medium and M2 macrophage medium. EdU-positive CMs are indicated with white arrows. *P < 0.05; n = 6 per group. (F-G) Immunofluorescence staining with pH3 of P1 and P7 CMs cultured in M1 macrophage medium and M2 macrophage medium. pH3-positive CMs are indicated with white arrows. *P < 0.05; n = 6 per group. (H-I) Immunofluorescence staining with Aurora B of P1 and P7 CMs cultured in M1 macrophage medium and M2 macrophage medium. Aurora B-positive CMs are indicated with white arrows. *P < 0.05; n = 6 per group.

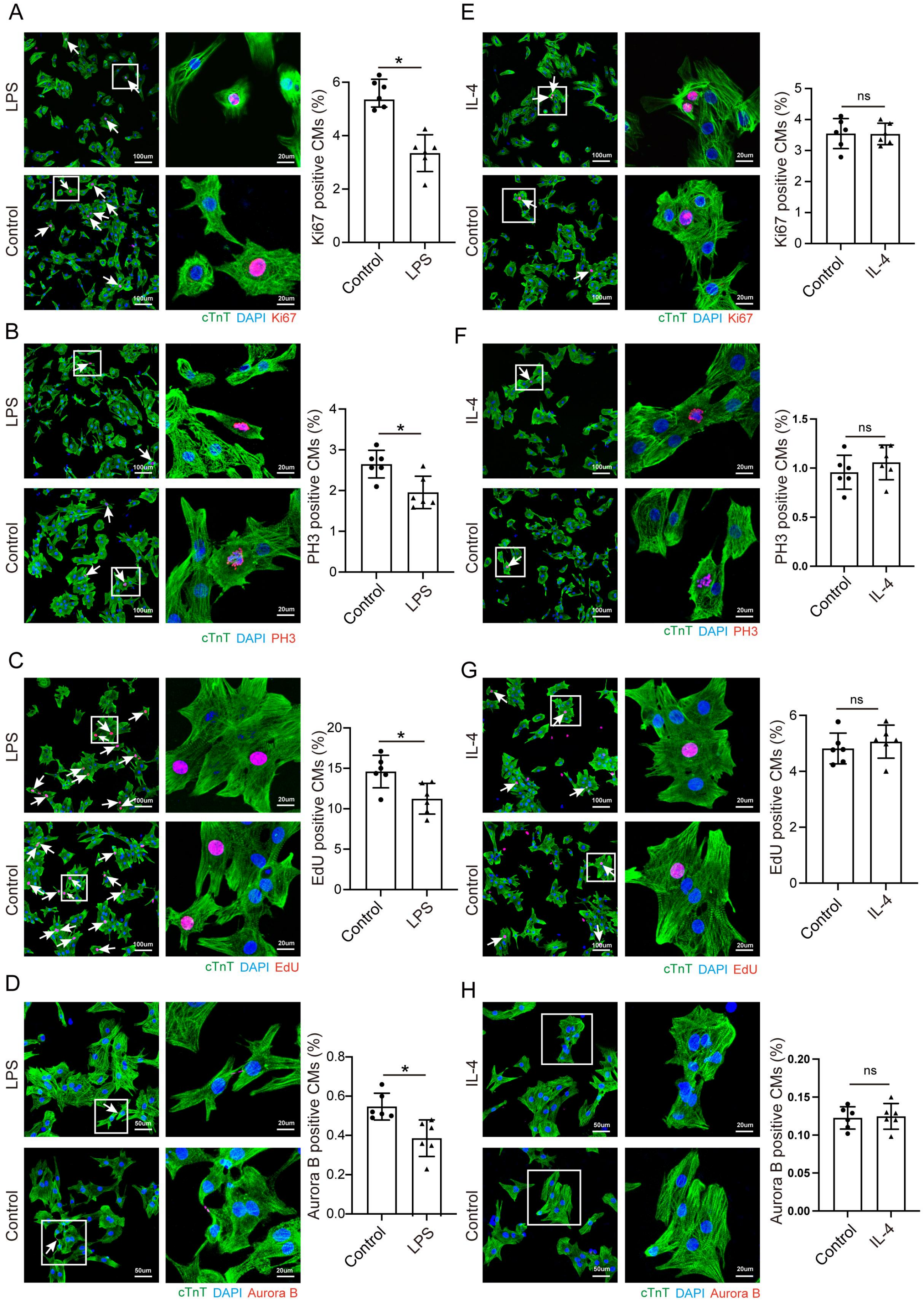
A
LPS
Control
100um
20um
Ki67 positive CMs (%)
*
0
2
4
6
Control
LPS
cTnT DAPI Ki67
B
LPS
Control
PH3 positive CMs (%)
*
0
1
2
3
4
cTnT DAPI PH3
C
LPS
Control
EdU positive CMs (%)
*
0
5
10
15
20
cTnT DAPI EdU
D
LPS
Control
50um
Aurora B positive CMs (%)
*
0.0
0.2
0.4
0.6
0.8
cTnT DAPI Aurora B
E
IL-4
Control
Ki67 positive CMs (%)
ns
0
1
2
3
4
5
IL-4
cTnT DAPI Ki67
F
IL-4
Control
PH3 positive CMs (%)
ns
0.0
0.5
1.0
1.5
cTnT DAPI PH3
G
IL-4
Control
EdU positive CMs (%)
ns
0
2
4
6
cTnT DAPI EdU
H
IL-4
Control
Aurora B positive CMs (%)
ns
0.00
0.05
0.10
0.15
0.20
cTnT DAPI Aurora B

57

Supplemental Figure 4 The effects of LPS and IL-4 themselves on CM proliferation

(A) Immunofluorescence images of isolated P1 CMs labeled with Ki-67 and cTnT. Quantification of Ki-67-positive CMs in the control and LPS-treated groups. (532 CMs from 5 neonatal mice in the control group and 432 CMs from 9 neonatal mice (P1) in the LPS-treated group). Ki-67-positive CMs are indicated by arrows. (B) Immunofluorescence images of isolated P1 CMs stained for pH3 and cTnT. Quantification of pH3-positive CMs in the control and LPS-treated groups. (401 CMs from 6 neonatal mice (P1) in the control group and 399 CMs from 6 neonatal mice (P1) in the LPS-treated group). pH3-positive CMs are indicated by arrows. (C) Immunofluorescence images of isolated P1 CMs stained with EdU and cTnT. Quantification of EdU-positive CMs in the control and LPS-treated groups. (665 CMs from 6 neonatal mice (P1) in the control group and 598 CMs from 5 neonatal mice (P1) in the LPS-treated group). EdU-positive CMs are indicated by arrows. (D) Immunofluorescence images of isolated P1 CMs stained for Aurora B and cTnT. Quantification of Aurora B-positive CMs in the control and LPS-treated groups (489 CMs from 10 neonatal mice (P1) in the control group and 512 CMs from 9 neonatal mice (P1) in the LPS-treated group). Aurora B-positive CMs are indicated by arrows. (E) Immunofluorescence images of isolated P7 CMs labeled with Ki-67 and cTnT. Quantification of Ki-67-positive CMs in the control and IL-4-treated groups (342 CMs from 7 neonatal mice (P7) in the control group and 412 CMs from 7 neonatal mice (P7) in the IL-4-treated group). Ki-67-positive CMs are indicated by arrows. (F) Immunofluorescence images of isolated P7 CMs stained for pH3 and cTnT. Quantification of pH3-positive CMs in the control and IL-4-treated groups. pH3-positive CMs are indicated by arrows. (G) Immunofluorescence images of isolated P7 CMs stained with EdU and cTnT. Quantification of EdU-positive CMs in the control and IL-4-treated groups (567 CMs from 7 neonatal mice (P7) in the control group and 552 CMs from 7 neonatal mice (P7) in the IL-4-treated group). EdU-positive CMs are indicated by arrows. (H) Immunofluorescence images of isolated P7 CMs stained with

Aurora B and cTnT. Quantification of Aurora B-positive CMs in the control and IL-4-treated groups (499 CMs from 9 neonatal mice (P7) in the control group and 476 CMs from 6 neonatal mice (P7) in the IL-4-treated group). Aurora B-positive CMs are indicated by arrows. ns, P>0.05 vs. the control group; bars = 100 μm and 25 μm, respectively, for Ki-67, pH3 and EdU staining; bars = 50 μm and 25 μm, respectively, for Aurora B staining.

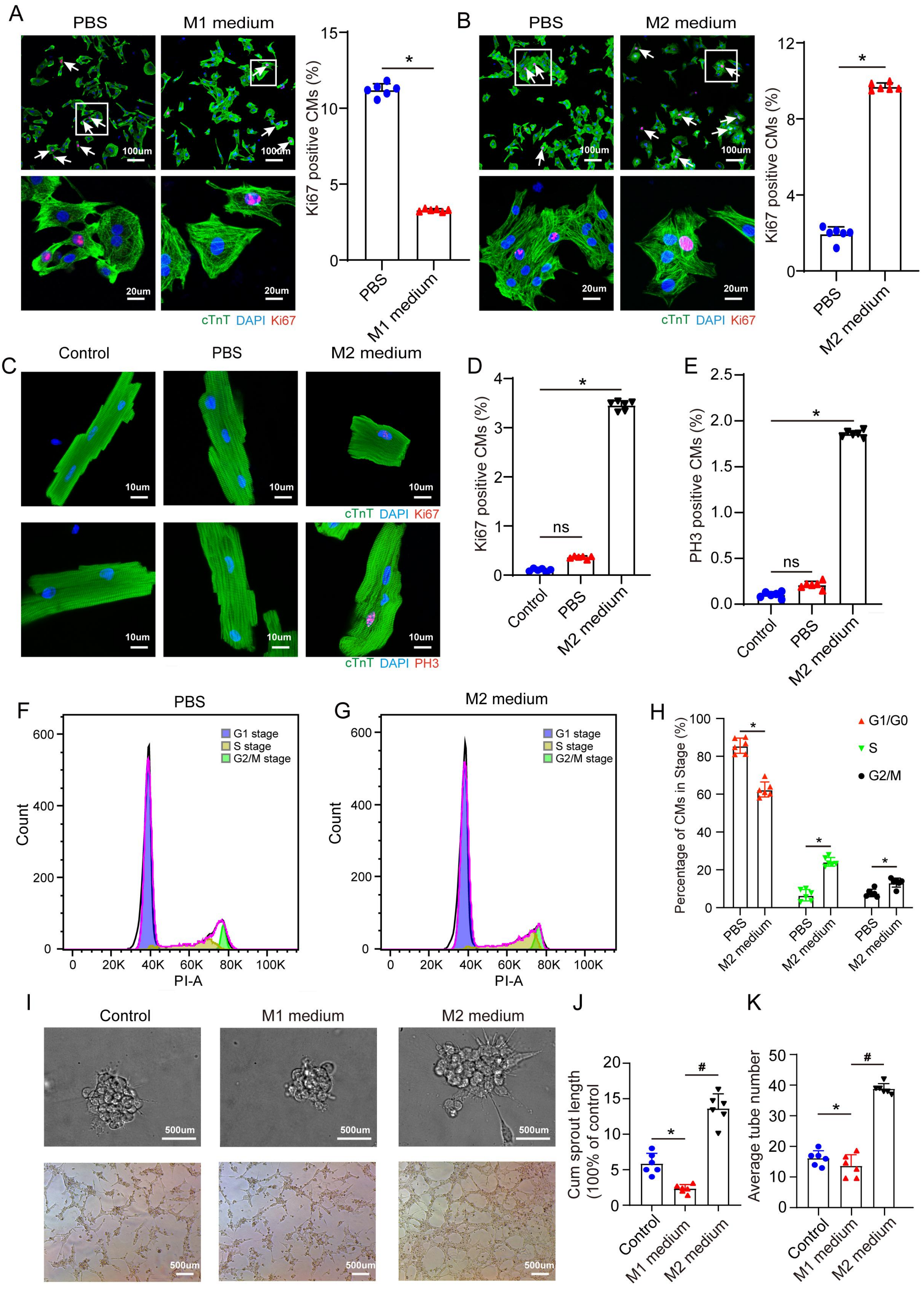
A
PBS
M1 medium
100um
20um
cTnT DAPI Ki67
Ki67 positive CMs (%)
PBS
M1 medium
B
PBS
M2 medium
100um
20um
cTnT DAPI Ki67
Ki67 positive CMs (%)
PBS
M2 medium
C
Control
PBS
M2 medium
10um
cTnT DAPI Ki67
cTnT DAPI PH3
D
Ki67 positive CMs (%)
ns
Control
PBS
M2 medium
E
PH3 positive CMs (%)
ns
Control
PBS
M2 medium
F
PBS
G1 stage
S stage
G2/M stage
Count
PI-A
G
M2 medium
G1 stage
S stage
G2/M stage
Count
PI-A
H
Percentage of CMs in Stage (%)
G1/G0
S
G2/M
PBS
M2 medium
I
Control
M1 medium
M2 medium
500um
J
Cum sprout length (100% of control)
Control
M1 medium
M2 medium
K
Average tube number
Control
M1 medium
M2 medium

86

Supplemental Figure 5 Activated macrophages induced CM proliferation and angiogenesis *in vitro*

(A-B) Immunofluorescence staining with Ki-67 of P1 and P7 CMs cultured in M1 macrophage medium and M2 macrophage medium. Ki-67-positive CMs are indicated with white arrows. (C-E) Immunofluorescence staining with Ki-67 of adult CMs cultured in M2 macrophage medium and negative control medium. $*P < 0.05$. (F-H) Flow cytometry analysis of P7 CMs cultivated with PBS or M2 medium. $*P<0.05$ vs. the PBS group; n=104 cTnT+ cells. (I) Upper panel: Representative images of vascular sprouting. Cells were cultured in M1or M2 medium. After 48 hours of continuous culture, HUVEC spheroids were allowed to sprout in a three-dimensional (3D) matrix for 24 hours (bars=500 μm). Lower panel: Matrigel-seeded HUVECs were activated with M1or M2 medium. After 24 hours in continuous culture, tube-like structures were detected. Statistical analysis of the mean number of tubes generated in each field was conducted (bars=500 μm). (J) Quantification of the total length of sprouts per spheroid. $*P<0.05$ vs. the control group; $\#P<0.05$ vs. M1 medium group. (K) Quantification of the mean number of tubes. $*P<0.05$ vs. the control group; $\#P<0.05$ vs. M1 medium group.

Supplemental Tables

Supplemental Table 1: Agents for pharmacological administration.

Name	Product number	Brand
Recombinant rat IL-4 (IL-4)	400-04	peprotech
Recombinant mouse beta-nerve growth factor (NGF)	C520464	Sangon Biotech
Lipopolysaccharides from *Salmonella enterica* serovar Typhimurium (LPS)	L6143-1MG	Sigma
Clodronate liposomes (from Liposoma B.V. Amsterdam) (CLD)	40337ES08	Yeasen
IL-10 Monoclonal Antibody (IL-10 antibody)	JES052A5	Thermo Fisher

Supplemental Table 2: Agents for AChE staining.

Name	Product number	Brand	CAS-No.
Agarose	A9045	Sigma	39346-81-1
Hyaluronidase	H1115000	Sigma–Aldrich	9001-54-1
tetrasodium diisopropylphosphoramide (also known as tetraisopropyl pyrophosphoramide)	T1505	Sigma	513-00-8
Sodium acetate	S2889	Sigma–Aldrich	127-09-3
Acetylthiocholine iodide	A5751	Sigma	1866-15-5
Sodium citrate	1613859	US Pharmacopeia	6132-04-3
Copper(II) sulfate pentahydrate	C8027	Sigma	7758-99-8
Potassium hexacyanoferrate(III)	P8131	Sigma–Aldrich	13746-66-2
Triton-X 100	CT11451	Coolaber	9002-93-1

Supplemental Table 3: Primary antibodies and secondary antibodies for immunofluorescence.

Primary antibodies

Name	Product number	Brand	Concentration
Anti-choline acetyltransferase	ab18736	Abcam	1:100
Anti-beta III tubulin	ab78078	Abcam	1:100
Anti-troponin T-C	sc-20025	SANTA CRUZ BIOTECHNOLOGY	1:50
Goat anti-wheat germ agglutinin (WGA)	AS-2024	Vector Laboratories	1:100
Anti-NGF	ab52918	Abcam	1:100
Anti-Ki-67	ab15580	Abcam	1:100
Rabbit monoclonal anti-phospho-histone H3 (Ser10) (pH3)	AF1180	Beyotime	1:100
Anti-Aurora B	ab2254	Abcam	1:100
Mouse monoclonal anti-CD18 (IB4)	217660	Millipore	1:100
Anti-alpha smooth muscle Actin antibody (anti- α - SMA)	ab150301	Abcam	1:100
Recombinant Anti-CD105 antibody (anti-CD105)	ab221675	Abcam	1:100
Arginase-1 Polyclonal Antibody (anti-Arg1)	16001-1-AP	proteintech	1:100
Anti-MAP2 antibody - Neuronal Marker (anti-MAP2)	ab32454	Abcam	1:100
Collagen III alpha 1/COL3A1 Antibody	NB600-594	Novus Biologicals	1:100
CD206 Polyclonal Antibody	18704-1-AP	proteintech	1:100

Secondary antibodies

Name	Product Number	Brand	Concentration
Donkey anti-sheep IgG H&L/Alexa Fluor 488	ab150177	Abcam	1:100

Goat anti-mouse IgG H&L/Alexa Fluor 647	bs-0296G-AF647	Bioss	1:100
Goat anti-mouse IgG H&L/Alexa Fluor 488	bs-0296G-AF488	Bioss	1:100
Mouse anti-goat IgG H&L/AF594	bs-0294M-AF594	Bioss	1:100
Goat anti-rabbit IgG H&L/Alexa Fluor 594	bs-0296G-AF594	Bioss	1:100

Supplemental Table 4: Primers for qPCR.

Name	Forward sequence	Reverse sequence
Ccnd2	AGACCTTCATCGCTCTGT	TGTGTTCACTTCATCATCCT
Cdk4	AGAGTGTGAGAGTTCCTAATG	GGTGTTGCGTATGTAGACT
Nrg1	TGGAGGATGAGGAATACGA	TCTTGGTTAGCGATTACACT
NGF	CACAGCCACAGACATCAA	CCTCTTCTTGTAGCCTTCC
Ach	ATTGTCAACCTCCTCATCC	ATCTCTGCCACCATTAGC

Supplemental Table 5: Primary antibodies for western blots.

Name	Product number	Brand	Concentration
Anti-M2 muscarinic acetylcholine receptor	M9558	Millipore Sigma	1:1000
Rabbit anti-GAPDH (loading control)	bs-2188R	Bioss	1:1000
Anti-NGF	ab52918	Abcam	1:1000
Anti-STAT3 (phospho-Y705)	ab76315	Abcam	1:1000
Rabbit monoclonal anti-phospho-Stat3 (Tyr705) (D3A7) XP®	9145	Cell Signaling Technology	1:1000
Polyclonal anti-beta-actin	20536-1-AP	Proteintech	1:1000
Anti-rabbit YAP	14074	Cell Signaling Technology	1:5000

Anti-rabbit Phospho-YAP (Ser127)	13008	Cell Signaling Technology	1:5000
Anti-phospho-Histone H3	06-570	Millipore	1:5000
Cyclin D1 Monoclonal antibody (anti-CyclinD1)	60186-1-Ig	Proteintech	1:2000
Cyclin D2 Polyclonal antibody (anti-cCyclinD2)	10934-1-AP	Proteintech	1:2000

Supplemental Table 6: Antibodies for flow cytometry.

Name	Product number	Brand	Concentration
PE anti-mouse F4/80	123110	BioLegend	1:50
FITC anti-mouse CD86	105006	BioLegend	1:50
APC anti-mouse CD206 (MMR)	141708	BioLegend	1:50

Figure1

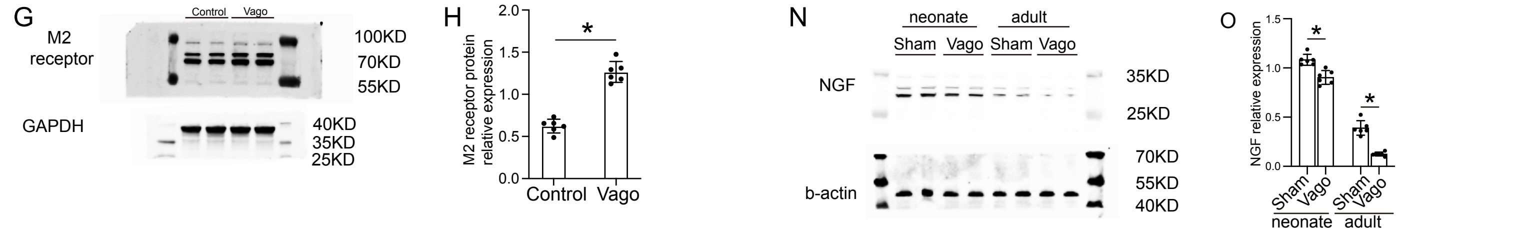

(G) Western blot of the M2 receptor level in control and vagotomized adult mice, showing upregulation of M2 receptor expression following vagotomy.

(H) Quantification of the M2 receptor data from panel G, p <0.05 vs. the control group; n= 6 per group.

(N) NGF protein levels detected by Western blotting after vagotomy in neonates and adult mice (b-actin was used as the internal reference).

(O) Quantification of protein levels according to panel N. p <0.05 vs. sham group. n= 6 per group.

Figure3

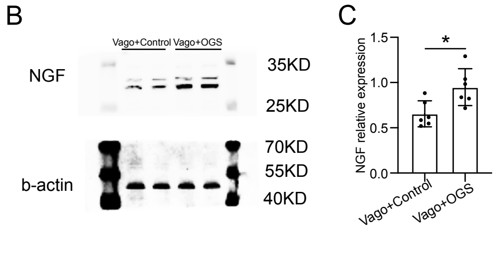

(B-C) NGF expression in the neonatal Vago+Control and adult Vago+ OGS groups. p<0.05 vs. the Vago+Control group.

Figure8

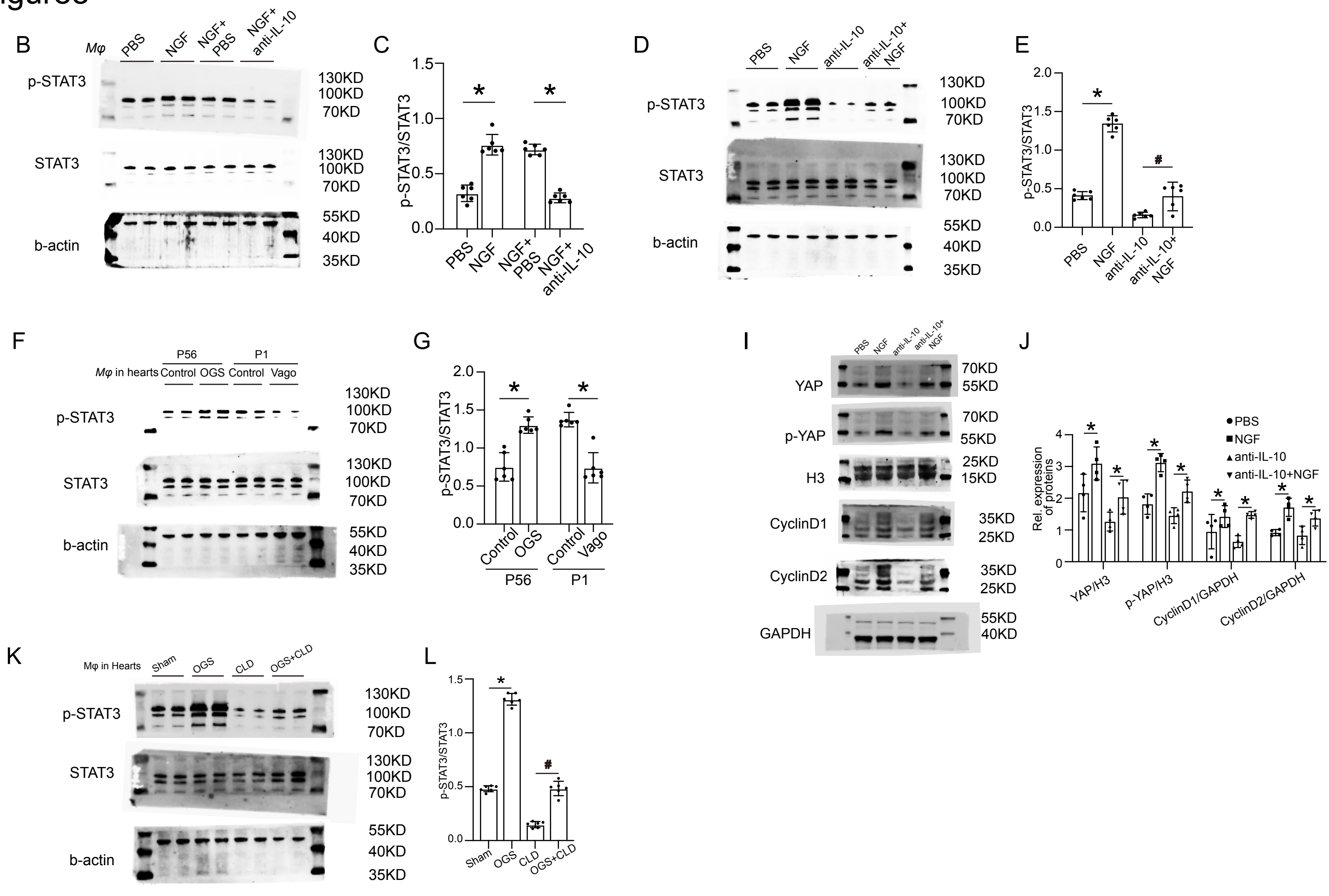

(B-E) Western blot detection of p-STAT3 and STAT3 protein levels in macrophages, along with corresponding statistical analysis results, after different agents aderministration in macrophages. P < 0.05; n = 6 per group.

(F-G) Protein expression levels of p-STAT3 and STAT3 in macrophages from corresponding cardiac tissues after the administration of GOS in infarcted adult mice, as well as after vagotomy in infarcted neonatal ones, and the results of their statistical analysis. P < 0.05; n = 6 per group.

(I-J) Western blotting and quantitative analysis of YAP, p-YAP, CyclinD1, and CyclinD2 protein levels in P7 CMs cultured with condition medium from macrophages treated with PBS, NGF, anti-IL-10, and anti-IL-10+NGF. P < 0.05; n = 4 per group.

(K-L) Western blotting and quantitative analysis of p-STAT3 and STAT3 protein levels in macrophages from different adult mice groups (Sham, OGS, CLD, and OGS+CLD). P < 0.05; n = 6 per group.
